# Effect of Omega-3 Fatty Acid Ethyl Esters on the Oxylipin Composition of Lipoproteins in Hypertriglyceridemic, Statin-Treated Subjects

**DOI:** 10.1371/journal.pone.0111471

**Published:** 2014-11-13

**Authors:** John W. Newman, Theresa L. Pedersen, Verdayne R. Brandenburg, William S. Harris, Gregory C. Shearer

**Affiliations:** 1 USDA, ARS, WHNRC, Obesity and Metabolism Research Unit, Davis, CA, United States of America; 2 Department of Nutrition, University of California Davis, Davis, CA, United States of America; 3 Family Medicine, Sanford School of Medicine, University of South Dakota, Sioux Falls, SD, United States of America; 4 Internal Medicine, Sanford School of Medicine, University of South Dakota, Sioux Falls, SD, United States of America; 5 Basic Biomedical Sciences, Sanford School of Medicine, University of South Dakota, Sioux Falls, SD, United States of America; 6 Cardiovascular Health Research Center, Sanford Research/USD, Sioux Falls, South Dakota, United States of America; 7 Department of Nutritional Sciences, The Pennsylvania State University, University Park, Pennsylvania, United States of America; University of Milan, Italy

## Abstract

**Background:**

Oxylipins mediate inflammation, vascular tension, and more. Their presence in lipoproteins could explain why lipoproteins mediate nearly identical activities.

**Methods:**

To determine how oxylipins are distributed in the lipoproteins of hypertriglyceridemic subjects, and whether omega-3 fatty acids alter them in a manner consistent with improved cardiovascular health, we recruited 15 dyslipidemic subjects whose levels of low density lipoprotein cholesterol (LDL-C) were at goal but who remained hypertriglyceridemic (200–499 mg/dL). They were treated them with the indicated dose of 4 g/d omega-3 acid ethyl esters (P-OM3) for 8 weeks. Measured oxylipins included mid-chain alcohols (HETEs, HEPEs and HDoHEs), ketones (KETEs), epoxides (as EpETrEs, EpETEs, and EpDPEs).

**Results:**

At baseline, arachidonate-oxylipins (HETEs, KETEs, and EpETrEs) were most abundant in plasma with the greatest fraction of total abundance (mean |95% CI|) being carried in high density lipoproteins (HDL); 42% |31, 57| followed by very low density lipoproteins (VLDL); 27% |20, 36|; and LDL 21% |16, 28|. EPA- and DHA-derived oxylipins constituted less than 11% of total. HDL carried alcohols and epoxides but VLDL was also rich in ketones. Treatment decreased AA-derived oxylipins across lipoprotein classes (−23% |−33, −12|, p = 0.0003), and expanded EPA−(322% |241, 422|, p<0.0001) and DHA-derived oxylipins (123% |80, 176|, p<0.0001).

**Conclusions:**

Each lipoprotein class carries a unique oxylipin complement. P-OM3 treatment alters the oxylipin content of all classes, reducing pro-inflammatory and increasing anti-inflammatory species, consistent with the improved inflammatory and vascular status associated with the treatment.

**Trial Registration:**

ClinicalTrials.gov NCT00959842

## Introduction

The control of LDL-C is effective in reducing cardiovascular disease (CVD; see list of abbreviations, File S3) [1]–[3], however other components of risk have yet to be effectively managed, including inflammation and impaired endothelial function. Fatty acids (FA) play an important role in these processes. Polyunsaturated FAs undergo modification into oxylipins, an important superclass of lipid mediators which includes the arachidonic acid (AA)-derived eicosanoids. These autacoids are primarily generated by one of three enzymatic pathways: cyclooxygenases (COXs) which generate the prostanoids and thromboids; lipoxygenases (LOXs) which generate leukotrienes, resolvins, hepoxilins and mid-chain alcohols; and cytochrome p450s (CYPs) which generate epoxides and omega-terminal alcohols [4].

Oxylipins are potent mediators of inflammation and vascular function, and their potencies can vary by parent FA [5]–[7]. While most *in vivo* studies have focused on AA-metabolites, we have shown that the administration of prescription omega-3 fatty acids (P-OM3) can reduce the abundance of oxylipins derived from AA, while increasing the abundance of eicosapentaenoate- (EPA) and docosahexaenoate- (DHA) derived oxylipins [8], [9]. Oxylipins are generally considered to act locally, however their presence in plasma suggests they act in an endocrine fashion as well. In rats, most oxylipins (>90% of those present in plasma) are found acylated in the glycerolipids of lipoproteins [10]. While the specific distribution of oxylipins within human lipoproteins has not been reported, in an animal model of chronic, inflammatory disease with dyslipidemia there is a shift of lipoprotein-oxylipin profiles in an inflammatory direction [11]. Further, the release of oxylipins by lipoprotein lipase from rat lipoproteins demonstrates they may be delivered to LpL-expressing tissues (adipose, heart, and skeletal muscle) esterified in circulating glycerolipids [10]. Oxylipins are known to be esterified in cellular phospholipids [12], [13], suggesting that lipoprotein phospholipids may also carry oxylipins.

Given the effects of P-OM3 on oxylipin profiles [8] and their concurrent reduction of CVD risk, serum TG, inflammation, and improvement in vascular function, it is plausible omega-3 fatty acids produce these effects by altering the quantity and quality of lipoprotein-oxylipins. In the current study we examine the impact of P-OM3, in the context of a statin-induced, maximal reduction in levels of low density lipoprotein cholesterol (LDL-C). We compared lipoprotein oxylipin composition in a change-from-baseline design in order to 1) document the distribution of oxylipins among lipoproteins in humans and 2) measure the effect of P-OM3 on the lipoprotein distribution of oxylipins, especially on certain pro- and anti-inflammatory, and on vasoactive oxylipins.

## Methods

### Participants & study location

The study was conducted by Sanford Research/USD in cooperation with physicians at Sanford Health. Our goal was to enroll subjects matching the inclusion/exclusion criteria used in the COMBOS trial [14] so our findings would reflect what occurred in that study. Accordingly, subjects on statin therapy with persistent hypertriglyceridemia were selected from the electronic medical records at Sanford Health and screened to ensure they met the COMBOS criteria (Figure S1 in File S1), then were contacted by letter and invited to participate. Responders were screened during a phone interview and, if qualifying, were sent to the study physician (VB) for a screening visit where clinical lipid and safety panels were drawn. Qualifying subjects returned one week later for a baseline visit after which they began P-OM3 therapy. They returned 63 days later for their final visit where compliance was assessed by pill count. Subjects were recruited from September 2009 to February 2010. The protocol was registered at clinicaltrials.gov (NCT00959842) and approved by the IRB at Sanford Research/USD and written informed consent was obtained prior to participation.

### Study design

This was designed as a simple open-label, change-from-baseline study. Treatment was for 8 weeks with 4 g/day prescription omega-3 acid ethyl esters (P-OM3; Lovaza, GlaxoSmithKline). This dose provided 1,860 mg of EPA and 1,500 mg of DHA for a total of 3.4 g omega-3 FAs/day as acid ethyl esters for a total of 3.4 g omega-3 FAs/day. The dose is indicated in the USA for triglycerides >500 mg/dL and has variation on levels and purposing in other nations as well. Since P-OM3 affects the composition and concentration of the lipoproteins themselves (most notably VLDL), the oxylipin concentration was normalized to lipoprotein phospholipid in order to highlight how each lipoprotein's composition was affected. Our objectives were to 1) characterize lipoprotein-oxylipin at baseline and generally characterize the inflammatory and vascular activities; 2) demonstrate the effect of P-OM3 therapy on the per phospholipid (PL) concentration of omega-3 oxylipins (EPA & DHA derived) and omega-6 oxylipins (AA derived) in HDL, LDL and VLDL as well as plasma. We also tested whether P-OM3 treatment: 3) increased the abundance of CYP-epoxygenase oxylipins over LOX oxylipins; 4) changed the abundance of oxylipins within the CYP-epxoygenase pathway as measured by the epoxide/diol ratio at each measured FA-double bond position; and 5) changed the abundance of oxylipins within the LOX pathway as measured by the mid-chain alcohol/ketone ratio at each measured FA-double bond position. Our interest was to investigate the plausibility of lipid mediators in mediating effects, not interventional efficacy. Thus, we operated under IND-exemption and specifically did not analyze by “intent-to-treat.” We measured the octadecanoic oxylipins (LA- and αLA-derived), but report these results in supplemental documents (since this study focused on AA-, EPA- and DHA-derived oxylipins.

### Plasma lipids

Plasma samples for lipoprotein composition were frozen in 20% sucrose. A single overnight spin recovered VLDL in a PBS fraction above the sucrose, and LDL in the sucrose gradient. HDL was further fractionated using FPLC on a Sepharose 6 10/300 GL column (GE Lifesciences, Upsala Sweden) [15]. Cholesterol, phospholipids, triglycerides and total proteins were measured using standard colorimetric assays, SDS-PAGE was used as a quality control [16].

### Fatty acids

The fatty acid composition of each lipoprotein fraction was measured as previously reported [17].

### Oxylipins

The esterified oxylipin composition of each lipoprotein fraction was determined as previously described for plasma [18].

### Statistical methods

Differences were assessed using mixed model ANOVAs adjusting for age and sex since these are known to affect lipoprotein outcomes. A natural logarithmic transformation was used to stabilize the variance and reduce skewness. Geometric means and 95% confidence intervals for baseline and follow-up were calculated for each treatment group. True effect size after accounting for age and sex are derived from the indicator parameter estimate and its standard error. Since logarithmic transformations were used, the effects are best understood as proportional, or percent, changes. If the interaction p-value was less than 0.2, the interaction was maintained in the model, otherwise it was dropped in order to obtain a pooled estimate of the treatment effect. JMP Pro 9.0.2 was used for statistical tests except where specified as using GraphPad Prism 6.01.

## Results

### Participant flow, baseline characteristics & blood lipids

A diagram representing subject flow is given in Figure S1 in File S1. Compliance was high, with 93% (91, 95) of pills administered taken on average; no subjects took less than 80%. In comparison to the COMBOS trial's active group, subjects in our study had lower serum TGs (282 *vs.* 237) and HDL-C (47 *vs.* 38) respectively [14] (Table 1). P-OM3 decreased plasma triglycerides by 49 (81, 17) mg/dL, VLDL-cholesterol by 9.7 mg/dL, and plasma phospholipids by 58 (90, 26) mg/dL. While oxylipins are incorporated into the PL, TG, and CE components of lipoproteins, those with 20+ carbons (eicosanoids and docosanoids) are primarily found in PL [13]. No survey has yet investigated oxylipin distribution among lipoprotein-lipids, however adjusting the data by fractional PL concentration resulted in the largest reduction in Aikake's corrected information criteria (AICc) for every oxylipin and lipoprotein fraction. Thus, oxylipins were normalized to PL using a molecular weight of 784 mg/mmol. The octadecanoids presented in Table S1 in File S2 should be interpreted cautiously since, based on unpublished findings in rodent models, we believe they are also prevalent in triglyceride and cholesterol esters. Adjustments for sex and age were rarely significant.

**Table 1:** Demographics and treatment effects on vascular and metabolic characteristics (N = 15).

			Change from baseline	
Y	Baseline^a^	Final^a^	Mean (95% CI)^b^	p-val
*Demographics*				
Age (yrs)	59 |46, 68|^c^	.	.	.
Female n (%)	5 (33%)	.	.	.
*Vascular*				
BMI	30.7 (28, 33)	30.6 (28, 33)	−0.03 (−0.44, 0.39)	0.89
BPM	72.6 (68, 77)	69.4 (65, 74)	−3.2 (0.017, −5.7)	0.68
Systolic	136 (130, 140)	136 (130, 140)	−0.1 (−6.2, 5.9)	0.96
Diastolic^L^	81.5 (77, 86)	80.3 (76, 85)	−1.5% (−4.7, 1.7)	0.32
*Metabolism (non-lipid)*				
Total Protein (gm/dL)^L^^,^ C	7.29 (7.1, 7.5)	7.06 (6.9, 7.2)	−3.1% (−5.9, −0.16)	0.04
Glucose (mg/dL)^L^^,^ C	106 (100, 110)	104 (98, 110)	−2.4% (−6.8, 2.2)	0.28
A1C (%) L ^,^ C	5.91 (5.6, 6.3)	5.99 (5.6, 6.4)	1.4% (−6.4, 9.9)	0.71
*RBC Fatty acid composition (weight %)*				
C18:2n6^L^	10.2 (9.3, 11.2)	9.8 (8.9, 10.7)	−4% (−8, 0)^d^	0.054
C18:3n6^L^	0.13 (0.12, 0.15)	0.12 (0.11, 0.14)	−16% (−41, 20)^d^	0.32
C18:3n3^L^	0.06 (0.04, 0.08)	0.05 (0.04, 0.07)	−6% (−16, 6)^d^	0.31
C20:3n6^L^	1.6 (1.3, 1.9)	1.4 (1.2, 1.6)	−13% (−19, −5)^d^	0.003
C20:4n6^L^	17 (17, 18)	15 (14, 16)	−13% (−15, −10)^d^	<0.0001
C20:5n3^L^	0.49 (0.41, 0.58)	1.90 (1.59, 2.27)	288% (214, 380)^d^	<0.0001
C22:6n3^L^	3.9 (3.5, 4.3)	6.4 (5.8, 7.1)	64% (50, 80)^d^	<0.0001
OMX-3^L^	4.4 (4.0, 4.9)	8.4 (7.5, 9.3)	90% (71, 112)^d^	<0.0001
*Lipid/lipoprotein parameters*				
Triglyceride (mg/dL)^C^	237 (200, 270)	187 (160, 220)	−49 (−81, −17)	0.005
Phospholipids (mg/dL)	307 (280, 330)	249 (220, 280)	−58 (−90, −26)	0.002
Cholesterol (mg/dL)^L^^,^ C	174 (160, 190)	167 (160, 180)	−3.8% (−11, 3.7)	0.29
VLDL-C (mg/dL)^C^	47 (41, 54)	38 (31, 44)	−9.7 (−16, −3.2)	0.007
LDL-C (mg/dL)^C^^,^ L	88 (78, 99)	90 (80, 100)	2.1% (−7.9, 13)	0.68
HDL-C (mg/dL)^C^^,^ L	38 (33, 42)	39 (35, 44)	4.4% (−1.8, 11)	0.16
Chol/HDL (ratio)^C^^,^ L	4.6 (4.1, 5.2)	4.3 (3.8, 4.8)	−7.8% (−14, −0.64)	0.04
TG/HDL (ratio)^C^^,^ L	6.0 (4.6, 7.9)	4.5 (3.4, 5.9)	−21% (−38, −11)	0.004

L Log-transformed to satisfy test assumptions.

C from clinical lipid panel.

a Least squares mean (95% CI).

b adjusted for age and sex.

c Median |Inter-quartile range|.

d denotes a relative change from baseline value, not an absolute change in (weight %).

### Plasma oxylipin distribution

**Figure 1:**
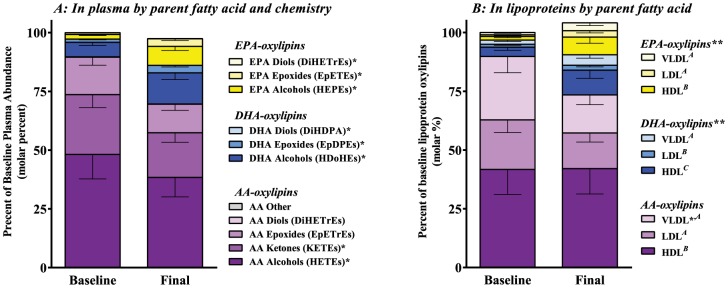
Distribution of oxylipins in plasma compartments. The distributions of oxylipins are expressed as a percent of the total baseline abundance before and after treatment. **A:** Oxylipins in plasma by parent fatty acid and chemistry, regardless of distribution in HDL, LDL, VLDL, or ‘free’. A mixed-model ANOVA was used for each FA. **B:** Oxylipins in lipoprotein by parent fatty acid, regardless of chemistry. A mixed-model ANOVA was used for each FA pool. n = 14: LDL was not recovered for one subject, making total calculations impossible. *p<0.05, **p<0.0005 versus baseline. Letters (*^A,B,C^*) indicate results of Tukey's HSD test for differences between lipoproteins. Those sharing a letter are not different.

Plasma oxylipins at baseline were primarily represented by AA-alcohols (HETEs), secondly by AA-ketones (KETEs), thirdly by AA-epoxides (EpETrEs), and then by EPA- and DHA-oxylipins (Figure 1). Since the change in AA-alcohols, AA-Ketones, and AA-epoxides was the same (p*_interaction_* = 0.93), the pooled estimate for reduction in AA-oxylipins was −23% |−33, −12|, p = 0.0003. Vicinal diols and other AA-oxylipins (not visible) followed the same trend however we did not formally test them since their lower abundance meant different variance. EPA pools were likewise uniformly expanded by 322% |241, 422|, p<0.0001, and DHA pools by 123% |80, 176|, p<0.0001; (EPA p*_interaction_* = 0.70 and DHA p*_interaction_* = 0.71). Treatment with P-OM3 reduced both the total and proportional abundance of AA-oxylipins, and increased the amount of EPA- and DHA-oxylipins.

Similar patterns emerged when oxylipins were analyzed by lipoprotein. Since the change in AA-oxylipins in HDL appeared to be distinct from that of VLDL (p*_interaction Visit × HDL_* = 0.07), we estimated the changes separately for each lipoprotein pool of AA-oxylipins. AA-oxylipins did not decrease in HDL 0% |−20, +17|, p*_unadjusted_* = 0.97, or in LDL −18% |−42, +2|, p*_unadjusted_* = 0.11, however they did decrease in VLDL by −15% |−28, −3|, p*_unadjusted_* = 0.02. EPA and DHA lipoprotein oxylipin pools were uniformly increased allowing for pooled estimates. EPA oxylipins increased by 308% |203, 450|, p<0.0001, and DHA increased by 125% |68, 203|, p<0.0001, regardless of lipoprotein. The complete set of oxylipins including regioisomers can be found in Table S1 in File S2.

### Fatty-acid alcohols & ketones at baseline

**Figure 2:**
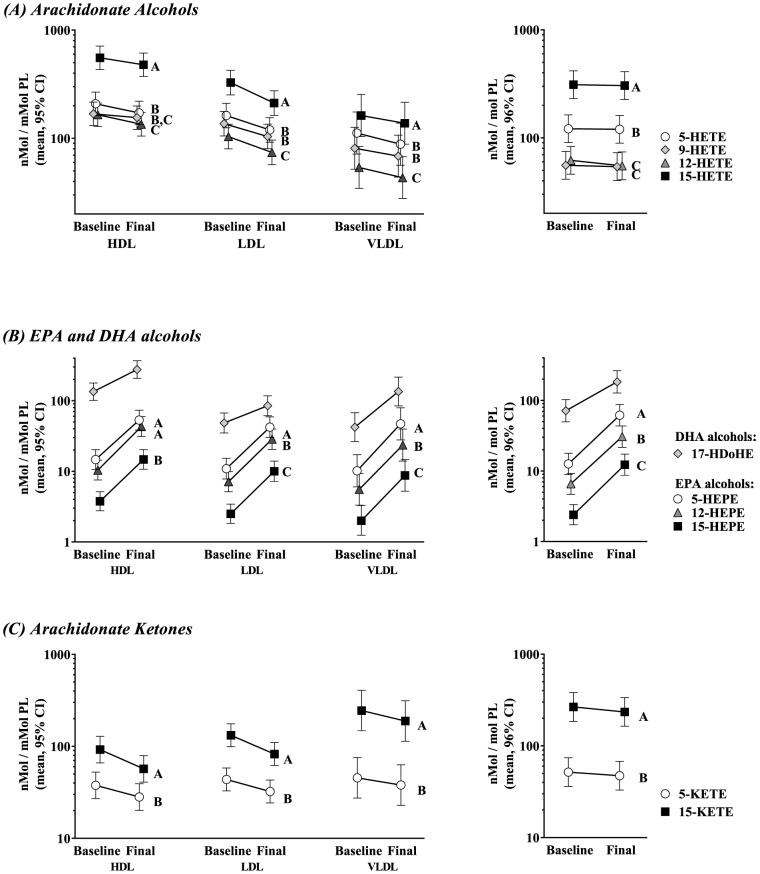
Distribution of fatty acid alcohols and ketones. The per-phospholipid concentration of mid-chain alcohols as AA-derived HETEs (**A**), EPA-derived HEPEs (**B**), DHA-derived HDoHEs (**B**) and AA-derived ketones (**C**) are shown in lipoproteins and plasma. Regioisomers are shaped and shaded by our best estimate of homologous double-bond positions across all figures. HETEs are mainly products of LOX, but are also products of CYP and autooxidation. KETEs are the HETE-dehydrogenase product of HETEs. Values for EPA and DHA ketones were not available. The effect of P-OM3 adjusted for age and sex was uniform regardless of regioisomer. The mean adjusted effects are shown graphically in Figure S2 in File S1; A mixed model ANOVA was used to test for differences on ln[nM oxylipin/mM PL]. The least-squares mean [95% CI] are shown. Tukey's HSD test was used for post hoc differences between regioisomer levels, and regioisomers sharing a letter are not different. Since no interactions were significant, the term was dropped and the parameter estimate was used to calculate a mean difference [95% CI]. Note that since the test was on log-transformed data, the effect is a proportional one (*i.e.* percent change). Note the log scale of the *y*-axis. Note that EPA and DHA oxylipins are shown on the same graphs for brevity, but constitute separate ANOVA tests. *^a^*9-HETE is an autooxidative product and is not formed by LOX.

The per-phospholipid concentration (nM oxylipin/mM PL) of AA, EPA and DHA mid-chain alcohols (HETEs, HEPEs, and HDoHEs respectively) at the baseline and final visits in HDL, LDL and VLDL are represented in Figure 2 and compared to plasma. P-OM3 did not affect the abundance of the regioisomers relative to each other (*e.g.* 5-HEPE to 15-HEPE). As previously reported [8], 15-HETE and 5-HETE were most abundant in plasma, but in lipoproteins 5-HETE was about as abundant as 9-HETE, an oxylipin that is not produced enzymatically and thought to be strictly an autooxidation product [19]. HDL contained the most HETEs per mole phospholipid and appears to represent the largest HETE pool in plasma. Among omega-3 alcohols, the DHA-derived 17-HDoHE was nearly as abundant as some HETEs in HDL and VLDL, but not in LDL. EPA- and DHA-alcohols both reflected the trend seen with HETEs, being most abundant in HDL followed by LDL then VLDL.

Mid-chain alcohols can be converted to ketones by HETE-dehydrogenase [20]. AA-ketones (KETEs) were present in each lipoprotein class in reverse order as alcohols, with VLDL>LDL>HDL (Figure 2).

### Fatty acid epoxides & vicinal diols at baseline

**Figure 3:**
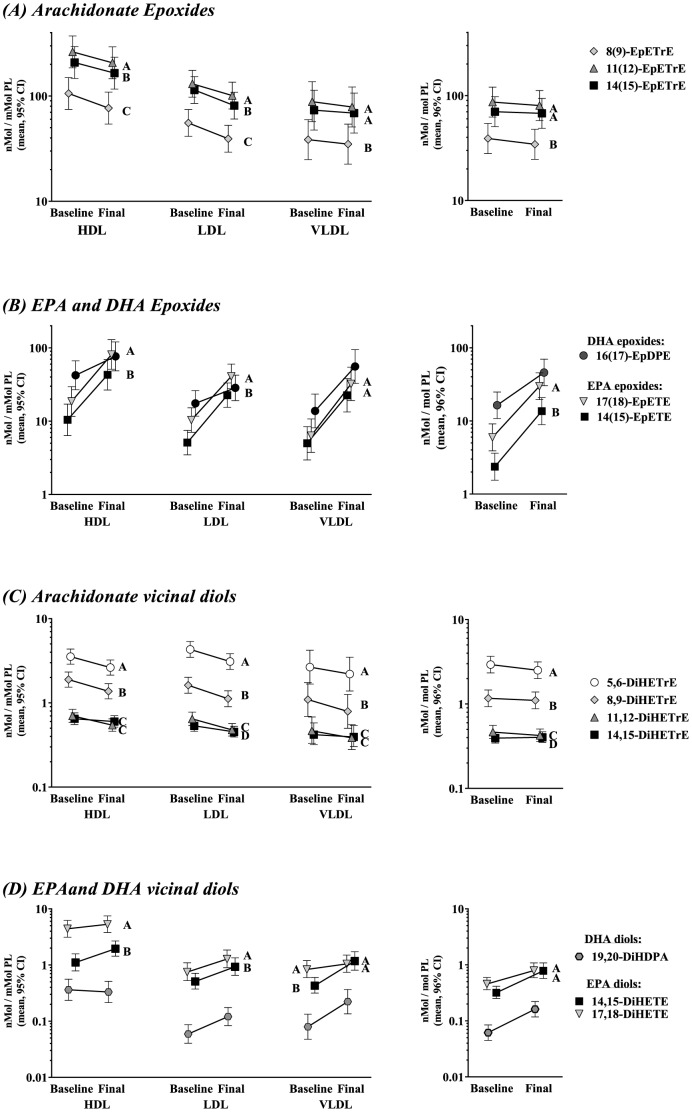
Distribution of fatty acids epoxides and vicinal diols. The per-phospholipid concentrations of epoxides as AA-derived EpETrEs (**A**), EPA-derived EpETEs (**B**), DHA-derived EpDPEs (**B**) AA-derived DiHETrEs (**C**), EPA-derived Di HETEs (**D**), and DHA-derived DiHDPAs (**D**) are shown in lipoproteins and plasma. Regioisomers are color coded by our best estimate of homologous double-bond positions across all figures. Epoxides are products of CYP-epoxygenase and can also be generated via autooxidation. Vicinal diols are the unique products of soluble epoxide hydrolase on epoxides. The effect of P-OM3 adjusted for age and sex was uniform regardless of regioisomer. The mean adjusted effects are shown graphically in Figure S2 in File S1; A mixed model ANOVA was used to test for differences on ln[nM oxylipin/mM PL]. The least-squares mean [95% CI] are shown. Tukey's HSD test was used for post hoc differences between regioisomer levels, and regioisomers sharing a letter are not different. Since no interactions were significant, the term was dropped and the parameter estimate was used to calculate a mean effect [95% CI]. Note that since the test was on log-transformed data, the effect is a proportional one (*i.e.* percent change). Note the log scale of the *y*-axis. Note that EPA and DHA oxylipins are shown on the same graph for brevity, but constitute separate ANOVA tests.

Fatty acid epoxides are produced by CYP-epoxygenases, but can also be autooxidatively generated. With the exception of the 5(6)-EpETrE, they are hydrolyzed by soluble epoxide hydrolase to produce vicinal diols. Epoxides were present at much higher levels than diols and were most abundant in HDL followed by LDL and VLDL. 14(15)- and 11(12)-EpETrE were the most abundant epoxides followed by the 8(9)-EpETrE (Figure 3). The DHA-derived 16(17)-EpDPE was the next most abundant, followed by EPA-epoxides. Among vicinal diols, the most abundant AA-diol was the 5,6-DiHETrE, decreasing in abundance with progression toward the ω-terminus, in reverse order as epoxide abundances. EPA diols were as, or more, abundant as AA-diols in many cases; the DHA-diol 19,20-DiHDPA was the least abundant.

### Treatment effects

The effect of P-OM3 treatment on lipoprotein and plasma oxylipins are summarized by chemical class and parent fatty acid in Figure S2 in File S1.

With one exception (VLDL-EPA-diols) the effect of treatment within fatty-acid and chemical class was the same, regardless of regioisomer. For both lipoproteins and plasma, there was a large increase in EPA and DHA oxylipins. EPA-alcohols and epoxides were increased from 300–400% of control, roughly equivalent to the increase in red blood cell EPA. EPA-diols were not increased to the same extent as EPA-alcohols or EPA-epoxides, and in HDL, DHA-diols were not increased at all. DHA-alcohols and -epoxides were increased in LDL and HDL to between 150% and 210% of baseline, however among VLDL this was 320% (157, 652) and 404% (207, 790) for alcohols and epoxides respectively, meaning DHA-epoxides were present in VLDL beyond the simple increase in RBC-DHA abundance. The effect of treatment among EPA and DHA-diols was more complex. DHA-diols in HDL were not increased at all by treatment, however in LDL they were increased by about the same amount as RBC DHA. In VLDL, DHA-diols were increased even more so. EPA-diols were increased less than the increase in RBC-EPA. In VLDL, an increase occurred in only one of the two regioisomers measured (17,18-DiHETE). P-OM3 also lowered AA oxylipins, particularly in HDL and LDL.

### Correlation of oxylipins with their parent (precursor) fatty acids by lipoprotein class

By demonstrating the association of oxylipins with their parent fatty acids, we help establish how amenable oxylipins are to precursor availability. Even at the baseline visit, EPA alcohols and epoxides were strongly related to erythrocyte EPA content, and this association persisted after treatment (Table S2 in File S2). DHA showed some similarities to EPA in association with DHA-oxylipins, however this dependence was reduced after treatment.

### Loss of correlation among oxylipin species by lipoprotein class

**Figure 4:**
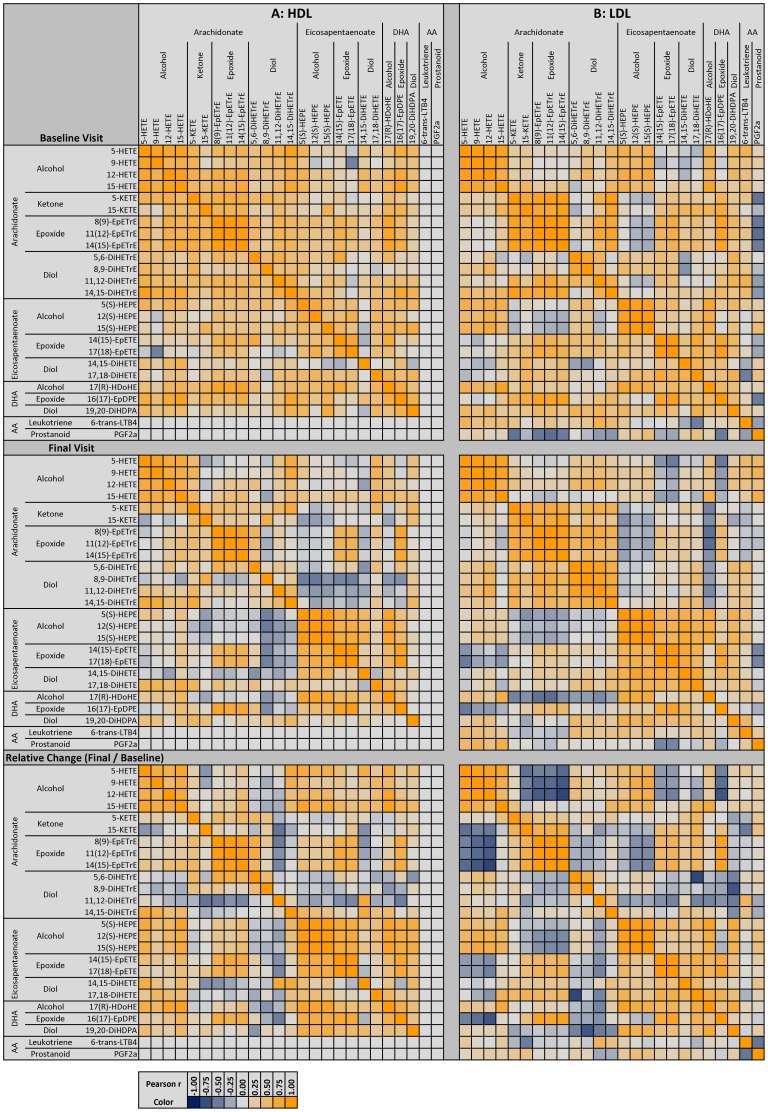
Correlation Heat Map for HDL and LDL. The Pearson correlation coefficient (r) among lipoprotein-oxylipins for HDL (**A**) and LDL (**B**) is shown before treatment **(top)**, after treatment **(middle)** as correlation of nM oxylipin/mM PL along with the change in oxylipins due to treatment **(bottom)** expressed as ln(nM oxylipin*_final_*/mM PL*_final_*)/ln(nM oxylipin*_baseline_*/mM PL*_baseline_*).

**Figure 5:**
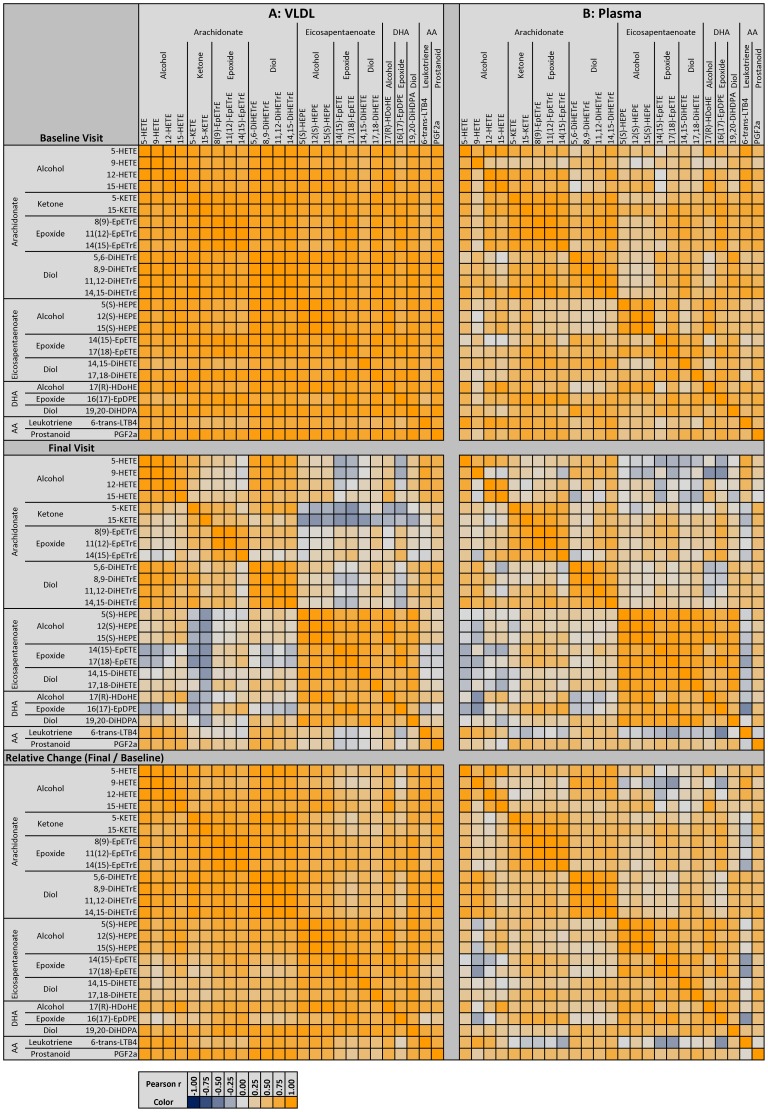
Correlation Heat Map for VLDL and whole plasma-oxylipins. The Pearson correlation coefficient (r) among lipoprotein-oxylipins for VLDL (**A**) and whole plasma oxylipins (**B**) is shown before treatment **(top)**, after treatment **(middle)** as correlation of nM oxylipin/mM PL along with the change in oxylipins due to treatment **(bottom)** expressed as ln(nM oxylipin*_final_*/mM PL*_final_*)/ln(nM oxylipin*_baseline_*/mM PL*_baseline_*).

The Pearson correlation matrices before and after P-OM3 treatment are presented in Figures 4 and 5. These matrices are useful in describing the independence of oxylipins species. At their baseline visit, the median |inter-quartile range| correlation among plasma oxylipins was 0.59 |0.39, 0.72|, a relatively strong interdependence of oxylipins, irrespective of parent FA or chemistry class. HDL and LDL oxylipins (Figure 4) were more independent; LDL oxylipins were the most independent (r = 0.32 |0.09, 0.55|), with nearly as much independence among HDL oxylipins, 0.44 |0.25, 0.59|. VLDL were the most interdependent, with strong correlations (r = 0.85 |0.77, 0.91| among nearly every oxylipin (Figure 5). P-OM3 treatment disrupted these interdependencies, demonstrated also by a shift in the distribution of correlation coefficients (Figure S3 in File S1). The effect was most pronounced among VLDL oxylipins, in which the correlations dropped by 0.52 to 0.33 |0.11, 0.66|, meaning oxylipins were less dependent one another and therefore reflective of more than one latent factor. In a similar manner, the median correlation also dropped among HDL and plasma oxylipins, to 020 |−0.03, 0.49| and 0.34 |0.11, 0.61| respectively, but dropped more moderately in LDL, by 0.08 to 0.24 |0.01, 0.49|.

### Comparison of LOX versus CYP pathway metabolites

The products of two major fatty-acid oxygenation pathways are prominently represented in the data; LOX and CYP. A formal means to test whether P-OM3 affects the relative activities is to test the effect of treatment on the ratio of epoxides to alcohols by lipoprotein and by modification position; for instance, an increase in the 11(12)-EpETrE to 12-HETE ratio can be interpreted as increased CYP-epoxygenase pathway activity relative to the 12-LOX pathway.

Among AA metabolites, the relative abundance of epoxides to alcohols was dependent upon the position of the modification and the lipoprotein (p*_interaction_* = 0.002). Specifically, there was no difference between plasma and any lipoproteins at the 11/12 position, however the abundance of 14(15)-EpETrE relative to 15-HETE was uniquely low in plasma compared to that in lipoproteins.

Among the metabolites of EPA, no differences in plasma and lipoproteins were present, however unlike AA metabolites, epoxides were overall more abundant than alcohols and so the pooled epoxide: alcohol ratio in lipoproteins was 2.62 (2.05, 3.35) compared to 0.77 (0.87, 0.93) for AA. This suggests that some biological process key to the appearance of epoxides in lipoproteins favors EPA, whereas AA is favored in producing alcohols. While EPA-epoxides were more abundant than alcohols at the “same” double bond (*i.e.* ω–*x* double bond; see Figure S4 in File S1) compared to AA-epoxides, further examination of this phenomenon revealed that the structural consistency was the number of vicinal double bonds; like the 14(15)-EpETE, which was the only epoxide∶alcohol measured in the EPA-oxylipin family, the AA-derived 11(12)-EpETrE has 2 vicinal double bonds and also outnumbers alcohols (Figure S4 in File S1). Among other FAs, alcohols outnumbered epoxides (*i.e.* the ratio <1). Treatment had no effect on the ratio for any FA family.

### Intra-pathway precursor/product ratios

As with comparisons of different pathways, within-pathway effects can be estimated using product∶precursor ratios. Here, effects on the CYP-epoxygenase pathway was measured using the diol∶epoxide ratio (Figure S5 in File S1) and effects on the LOX pathway as ketone∶alcohol ratios (Figure S5 in File S1). Treatment had no effect on arachidonate diol∶epoxide ratios, which were especially low at the 14/15 and the 11/12 position. Treatment increased the diol∶epoxide ratio at the 14,15 position of EPA by 84% |48, 130| and by 152% |101, 217| at the 17,18 position. Further, AA-epoxides were most abundant in HDL compared to plasma and other lipoproteins; among EPA-epoxides it was either the same (14/15 position) or reduced (17/18 position).

The distribution of alcohols and ketones among lipoproteins was more complex than with CYP pathway products and treatment had no effect. Among metabolites at the 5 position, HDL had the highest relative abundance of alcohols, followed by LDL then VLDL and plasma. Metabolites at the 15 position were more varied among lipoproteins, with HDL having a higher relative abundance of alcohols, followed by LDL, then plasma, then VLDL, in which ketones actually outnumbered alcohols.

## Discussion

The transport of oxylipins by lipoproteins has profound mechanistic implications since it reveals a non-local source of lipid mediators that could exert an effect on tissues. Here we used a targeted lipidomic investigation of lipoprotein oxylipins to demonstrate abundant, pro-inflammatory oxylipins in lipoproteins at baseline. Based on the general activity of the various oxylipins, we also demonstrate redistribution toward a less inflammatory profile in response to treatment with omega-3 FAs. Notably, this was observed in subjects with LDL-C at NCEP goals and whose LDL-C was unaffected by P-OM3.

This is the first lipidomic characterization of human lipoprotein-oxylipins. At baseline, VLDL, LDL, and HDL each have unique, characteristic oxylipins. Among LOX pathway products, HDL is enriched in mid-chain alcohols while VLDL is enriched in ketones. These lipoproteins could thus deliver LOX metabolites [10], [21] to their tissue targets, bypassing the local requirement for LOX activity. If true, it has implications for transcellular biosynthesis of 5-LOX metabolites such as leukotrienes and ketones. Similar activity has been noted [22] however a role for lipoproteins in delivering intermediates has not. The different composition of VLDL compared to HDL indicates each fraction would deliver different intermediates, and so have different actions: by delivering mid-chain alcohols, HDL could bypass the specific requirement for 5-LOX activity; however by delivering ketones, VLDL could additionally bypass the requirement for HETE-dehydrogenase and directly activate the OXE-receptor (*i.e.* TG1019, R527 and hGPCR48) [23]. Among LOX pathways, 5-LOX is known for its pro-inflammatory effects and 5-HETE, the AA product, is inflammatory and pro-atherogenic [24]. The same may not be true of its EPA homolog 5-HEPE, which has insulin sensitizing properties in animal models [25]. The difference in activities illustrates one means by which increasing the abundance of EPA oxylipins could have beneficial effects, especially when doing so at the expense of the AA homolog. The bioactivities of other LOX pathways are more difficult to summarize because their effects are less consistently pro- or anti-inflammatory. 12-LOX is generally considered pro-inflammatory, with activities such as mediation of endoplasmic reticulum stress and IL-12 secretion in adipocytes by 12-HETE (a 12-LOX product) [26], it may also have beneficial effects [27]. Unfortunately, a more pragmatic reason such comparisons can't be made is that in most cases only AA-metabolites have been studied. This makes it difficult to predict the effect of increasing EPA and DHA homologs.

A second major pathway covered is the CYP-epoxygenase pathway, whose fatty acid epoxides have potent vaso-protective activities, including protection from both septic [28] and hypertensive vascular diseases [29]. Fatty acid epoxides act on resistance vessels to produce an endothelial hyperpolarizing effect [30], and EC_50_ estimates are available in the same model for AA-, EPA- and DHA-epoxides [31], [32]. These studies offer a means to estimate the impact of P-OM3 treatment. Table 2 shows the hypothetical vasodilatory capacity of each lipoprotein, indicating that the benefit of P-OM3 treatment on measured epoxides is uniquely increased in VLDL. Imputing values and activities to unmeasured regioisomers (Table S3 in File S2) based on biochemical relationships observed in prior work [33] yields similar results. The former, empirical approach (Table 2) indicates an increased vasodilatory capacity among VLDL-epoxides, despite a 20% decrease in serum TG (*i.e.* VLDL). The imputed approach indicates increases in HDL and LDL, but again, the largest increase is in VLDL. Vasorelaxation is impaired in subjects with well controlled LDL-C and persistent hypertriglyceridemia [34], and VLDL contribute to the impairment [35]. Omega-3 FAs do not affect blood pressure in normotensive subjects, but induce a 7 mm Hg decrease in mean arterial when blood pressure is carefully measured (*i.e.* 24-hour ambulatory monitoring) in Stage 1 hypertensives [36]. More generally, they improve endothelial function in conjunction with lower serum TG [37], [38]. Given the correspondence between the effects of epoxides on vasodilation and the effects of omega-3 FAs on blood pressure, VLDL-epoxides seem a plausible explanation for this effect, but without specifically enquiring the VLDL-oxylipin pool, this relationship might not appear in plasma and be missed.

**Table 2:** Estimation of changes in lipoprotein-epoxide bioactivity.^a^

	Net potency for vasodilation^b^							
	HDL		LDL		VLDL		Plasma	
	Bs	Final	Bs	Final	Bs	Final	Bs	Final
Net AA [*^32^*]	0.99	0.77	0.52	0.38	0.34	0.31	0.33	0.31
Net EPA [*^32^*]	0.09	0.37	0.05	0.19	0.03	0.15	0.03	0.13
Net DHA [*^31^*]	0.07	0.14	0.03	0.05	0.02	0.10	0.03	0.08
Total	1.15	1.27	0.59	0.62	0.40	0.56	0.39	0.52
Percent-change		+11%		+4%		+42%		+34%

a unmeasured epoxides not included in calculation. Number of unmeasured epoxides: AA = 1, EPA = 3, DHA = 6. A table accounting for bioactivities of unmeasured epoxides by imputation is included in Table S3 in File S2.

b Net potency measured as Σ -*EC_50_×*[*Epoxide*]*_measured_* as reported by Ye *et al* & Zhang *et al*.

*Note*: potencies are measured in nM^2^/mM PL. Abbreviation: Bs – Baseline.

The global impact of P-OM3 treatment on lipoprotein-oxylipins is indicated by the decreased interdependencies. Greater interdependency, by definition, means less independence among oxylipin associated signaling pathways, and consequently a constrained system. At baseline, oxylipins within each lipoprotein class were very interdependent as indicated by high correlations, most extremely so among VLDL, but also among HDL, LDL and plasma in whole. While a major effect of P-OM3 was to increase nearly every omega-3 oxylipin regardless of lipoprotein class, P-OM3 also reduced the covariance of lipoprotein-oxylipins as well. Correlations within chemistry and parent fatty acid groupings remained strong after treatment, however correlation between oxylipins of different chemistry or parent FAs were weakened, indicating increasing flexibility of the system's bioactivity. Increased independence was most notable among VLDL- and HDL-oxylipins, and less so among LDL-oxylipins. Since the subjects here are already treated to near optimal LDL-C levels, it is unknown whether this is a natural feature of LDL, or a consequence of statin therapy. Surprisingly, only EPA abundance was strongly related to the observed abundance of EPA derived oxylipins, suggesting that factors other than simple precursor abundance are also responsible for the presence of oxylipins.

P-OM3 induced changes consistent with reduced inflammatory stress and a more flexible response to new stressors but the well known destinations of the lipoproteins themselves must also inform analyses since the destinations of lipoprotein-oxylipins reflect their parent lipoprotein. Thus, a complete understanding will require leveraging knowledge of oxylipin biology with that of lipoprotein-lipid trafficking. Most simplistically, this means hypothesizing directional transport of VLDL-oxylipins to peripheral tissues *via* lipoprotein lipase or receptor-mediated uptake, implying the myocardium, skeletal muscle and adipose are the primary recipients of VLDL-oxylipins. Conversely, for HDL it means peripheral acquisition of oxylipins from tissues by PL and cholesterol uptake followed by reverse transport to hepatocytes. Such an approach would go far in explaining the high abundance of pro-inflammatory oxylipins in HDL, a lipoprotein well known for its vaso-protective, anti-inflammatory effects: given HDL removal of oxylipins from peripheral tissues, the higher levels of HDL-HETEs and HDL-HODEs are consistent with more removal from peripheral tissues, and so provide a basis for the anti-inflammatory effect of HDL. Prior evidence in VLDL-oxylipins are consistent. The release of oxylipins from VLDL by LpL combined with the effect of lipolysis products on endothelial inflammation [21] support the use of lipoprotein lipid trafficking as a guide [10]. Further, unique bioactivities of lipoprotein lipolysis products by various LpL-family lipase/lipoprotein combinations on PPARs [39]–[42] suggest further study of each lipid pool (*i.e.* PL, cholesterol, TG, *etc.*) would increase the fidelity of findings.

### Limitations

This was a change from baseline study, meaning that we did not control for regression-to-the-mean effects by use of a placebo group. We attempted to control for this effect by requiring two prior high TG samples and a qualifying plasma sample with high TGs. In the COMBOS trial, TG in the placebo group was 3.5% lower after treatment, compared to a 49% decrease with active treatment in this study, suggesting the regression to the mean effect is small for lipoprotein lipids. While regression-to-the-mean effect sizes for oxylipins are unknown, prior studies indicate they are small however we cannot rule this out. As products of polyunsaturated fatty acids, it is possible that saturation of fatty acids in the acceptor lipid pools limits the impact of P-OM3, especially on EPA- and DHA-oxylipins.

## Conclusion

The oxylipin composition of each lipoprotein is unique, dependent on parent fatty acid and generating enzymatic pathways. Omega-3 fatty acids reduce pro-inflammatory species (*i.e*. HETEs) and increase anti-inflammatory species (*i.e.* EpETEs). These findings demonstrate a plausible mechanism for reducing cardiovascular risk by modulating lipoprotein-oxylipins.

## Supporting Information

Checklist S1 **Trend checklist.**
(PDF)

File S1 **Supporting figures.**
(PDF)

File S2 **Supporting tables.**
(PDF)

File S3 **Supplemental list of abbreviations.**
(DOCX)

Protocol S1 **Study protocol.**
(PDF)
