# Supplementary material for: Effect of Omega-3 Fatty Acid Ethyl Esters on the Oxylipin Composition of Lipoproteins in Hypertriglyceridemic, Statin-Treated Subjects
Source: PLoS One. 2014 Nov 13;9(11):e111471. doi: 10.1371/journal.pone.0111471 (PMC4230929; doi:10.1371/journal.pone.0111471)
Supplement: File S1 — Supporting figures. (PDF) [file pone.0111471.s002.pdf]

**Supplemental Figures:**
**Supplemental Figure 1: Participant flow**

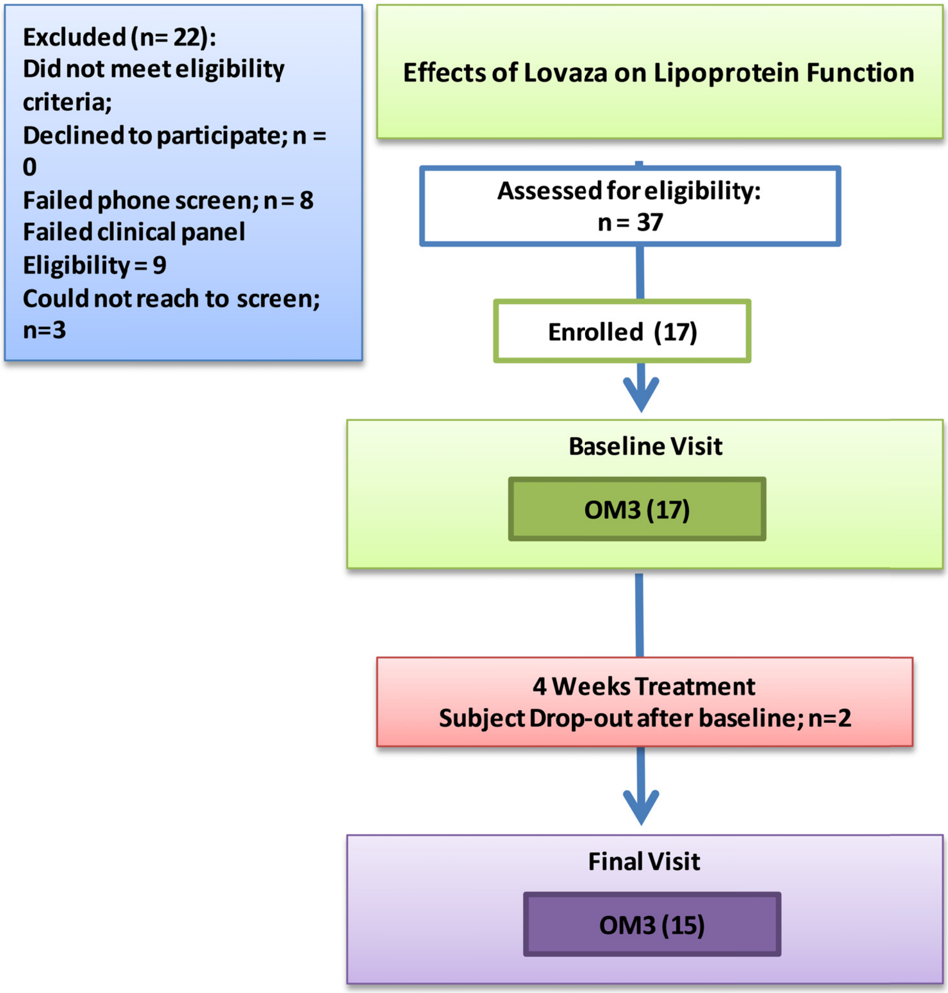

**Supplemental Figure 2: P-OM3 effect sizes**

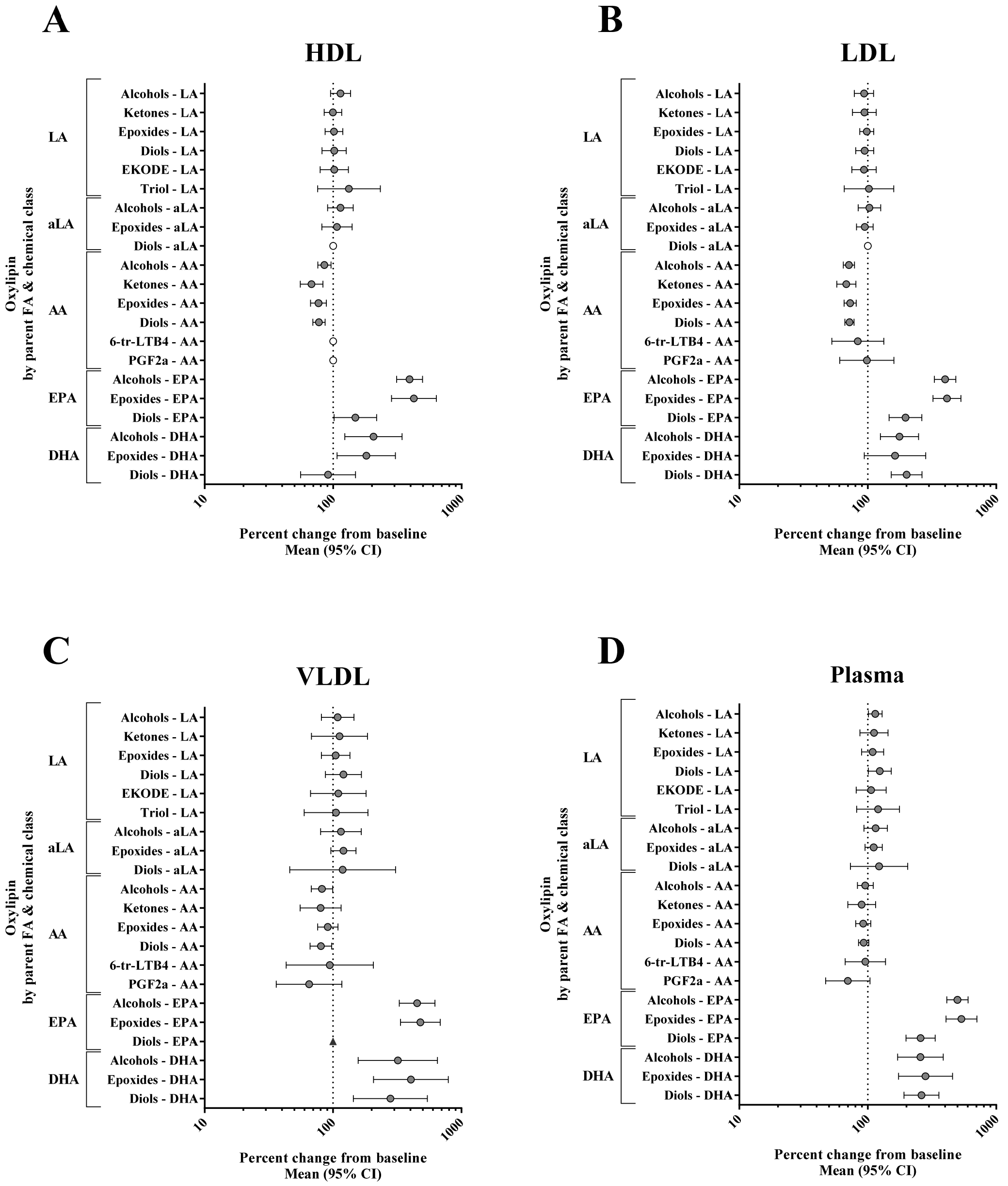


**Supplemental Figure 2: P-OM3 effects.** The effect of P-OM3 is present after adjusting for others (age and sex). The adjusted effect [95% CI] is calculated from the regression equation to express the change as a percent of baseline. The adjusted effect is given for HDL **(A)**, LDL **(B)**, VLDL **(C)**, and plasma **(D)**.

**Supplemental Figure 3: Distributions of Pearson Correlations at Baseline and Final Visits**

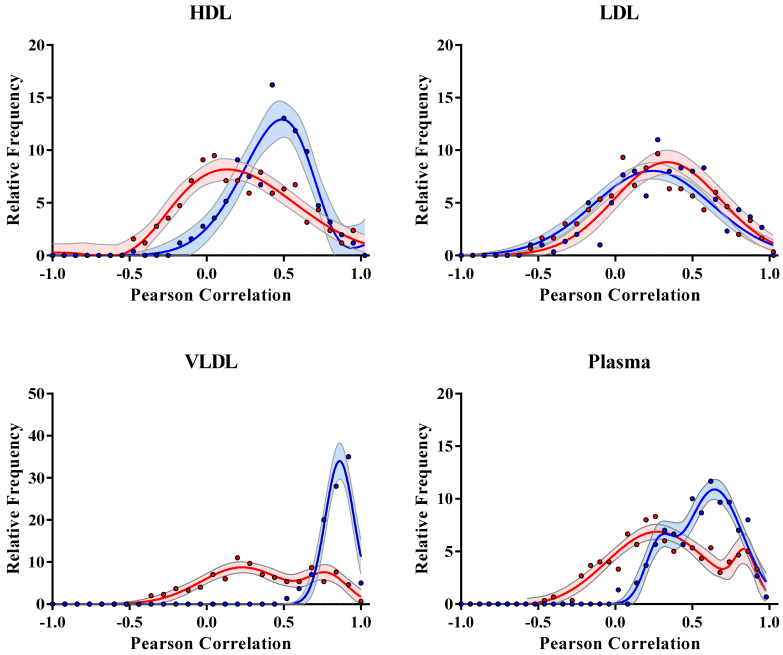


**Supplemental Figure 3: Distribution of correlations at baseline and final visits of correlation coefficients.** In order to visualize how correlations change in heat maps from figure 4, this figure graphs the distributions of all coefficients at the baseline and final visit. Each distribution was fitted using non-linear regression to Gaussian distributions that best described the data using AICc. The fitted line [95% confidence band] is shown. In each case, a double Gaussian distribution best fit the data, however there are clear shifts in correlation from baseline to final visits.

**Supplemental Figure 4: Relative abundance of epoxides and alcohols**

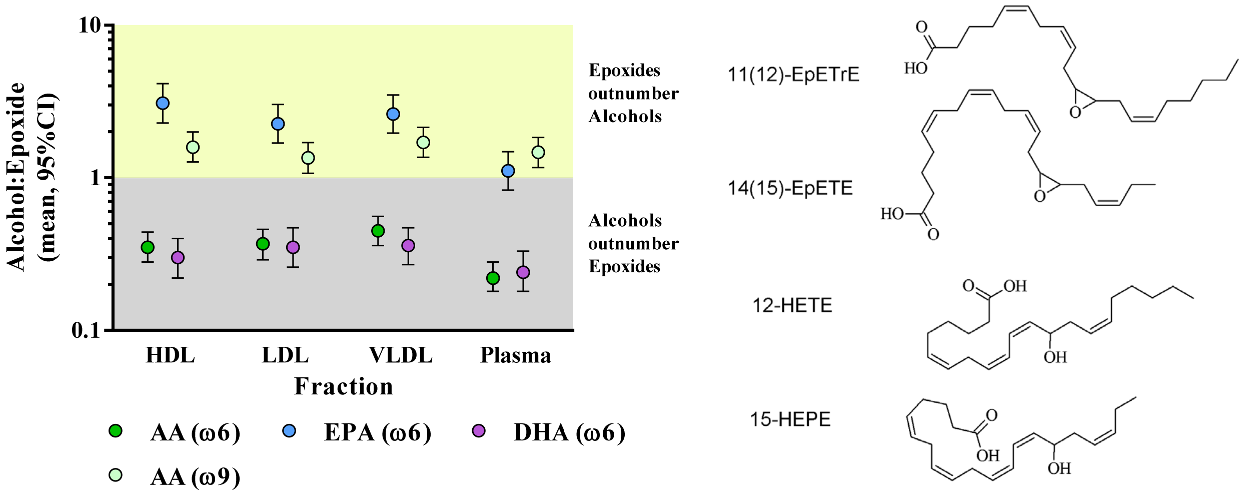


**Supplemental Figure 4: Relative abundance of epoxide and alcohols.** Given bioactivity of epoxides as normotensive, anti-inflammatory and the activity of alcohols and ketones as generally pro-inflammatory, the relative abundance of metabolites of each pathway provides an estimate how each lipoprotein-oxylipin pool might impact target tissues. Since the ratios are based on molar abundance, a ratio above 1 indicates that epoxides outnumber alcohols, (indicated by yellow background), while ratios below 1 indicate the opposite (grey). The abundance of epoxides did not change between lipoproteins, instead they were a function of parent fatty acid and/or the oxygenated double bond (structures given at right). For EPA, epoxides (EpETEs) at the ω6-double bond (*i.e.* 14(15)-EpETE) outnumbered the mid-chain alcohols (HETEs) at the same location (*i.e.* 15-HEPE). This was not the case for either the AA-epoxides ω6-double bond where 15-HETE outnumbered the 14(15)-EpETrE, or the DHA-epoxides at the ω6-double bond where the 17-HDoHE outnumbered the 16(17)-EpDPE. Interestingly, for the epoxides and alcohols at the AA ω9-double bond (*i.e.* 11(12)-EpETrE and 12-HETE respectively), the epoxides outnumbered the alcohols as with EPA. This is revealing, since the number of vicinal double bonds appears to predict the prevalence of epoxides rather than the apparent homologous position. The ratios are robust since they are calculated within the same sample and do not rely on other adjustments. A mixed model ANOVA was used to test for differences on ln[(nM epoxide$_{double\ bond\ a}$ / nM mid-chain alcohol$_{double\ bond\ a}$]. The least-squares mean [95% CI] from the combined baseline and final visit are shown since there was not effect of treatment. Note the log scale of the *y*-axis.

## Supplemental Figure 5: Diol:Epoxide ratios

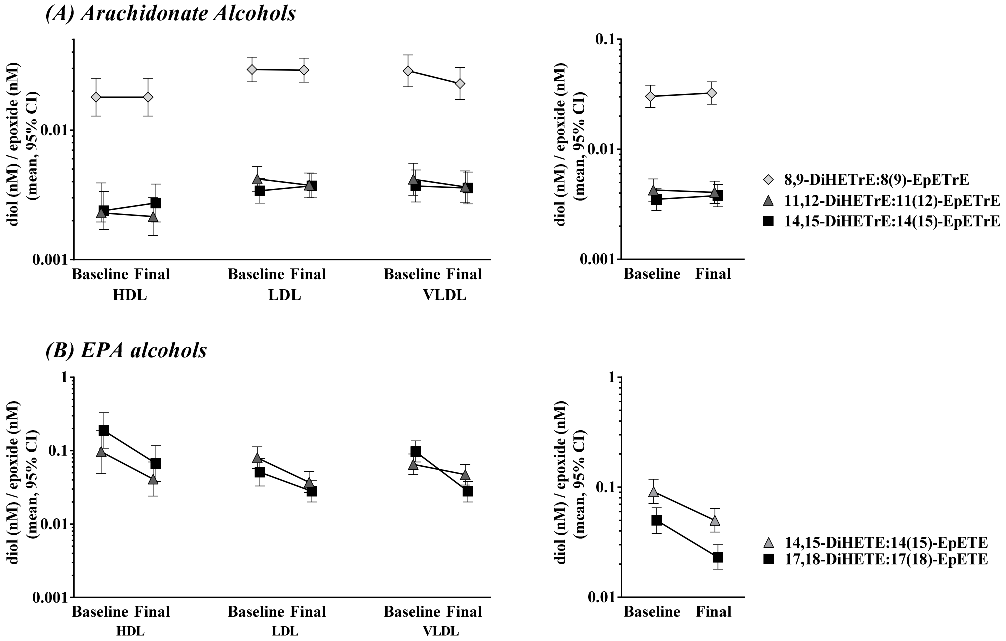


**Supplemental Figure 5: vicinal Diol:Epoxide ratios.** The relative abundance of vicinal diols to epoxides provides a biomarker for metabolic activity in the CYP-epoxygenase pathway. Since they are calculated within the same sample and do not rely on other adjustments the ratios are robust, and in all cases epoxides greatly outnumbered vicinal diols. P-OM3 did not affect ratios of AA metabolites, but decreased the ratio among EPA metabolites, possibly indicating increased epoxide generation. The relative abundance of epoxides did not vary greatly by lipoprotein but did by the double-bond on the parent FA. A mixed model ANOVA was used to test for differences on ln[(nM vicinal diol$_{double\ bond\text{-}x}$ / nM epoxide$_{double\ bond\text{-}x}$]. The least-squares mean [95% CI] are shown. Note the log scale of the *y*-axis.

**Supplemental Figure 6: Ketone:Alcohol ratios**

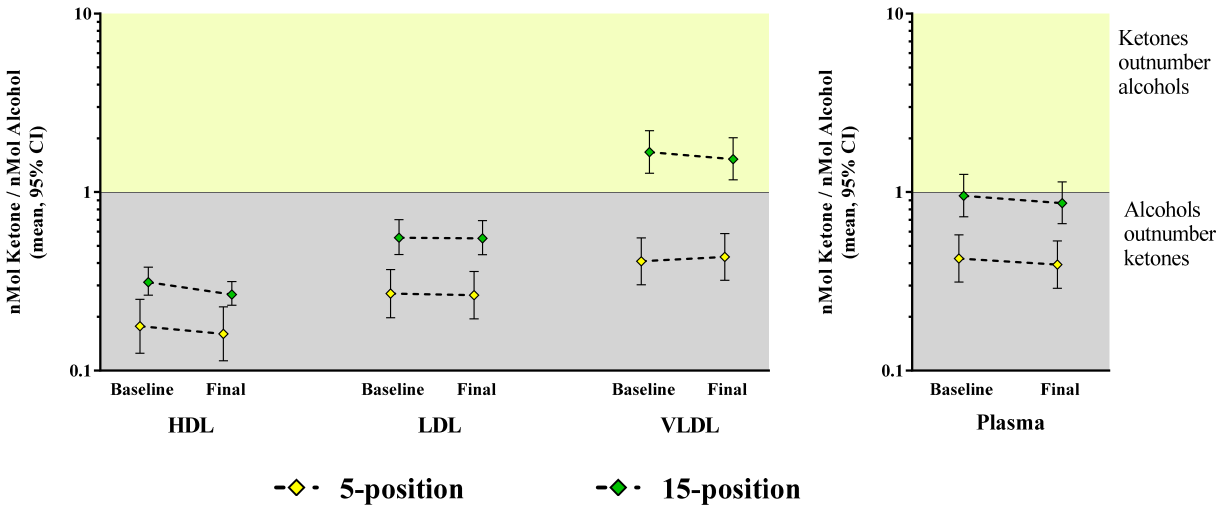


**Supplemental Figure 6: Ketone:Alcohol ratios.** The relative abundance of ketones to alcohols provides a biomarker for metabolic activity in the LOX pathways. Since they are calculated within the same sample and do not rely on other adjustments the ratios are robust, and in most cases alcohols outnumbered ketones. The abundance of ketones increased from HDL to LDL to VLDL, demonstrating a qualitative difference in the types of oxylipins that each lipoprotein class transports, possibly a function of the increasing neutral lipid content of lipoproteins as ketone abundance increases. No significant effects of P-OM3 were detected. A mixed model ANOVA was used to test for differences on ln[(nM $oxylipin_{final}$ / mM $PL_{final}$) / (nM $oxylipin_{baseline}$ / mM $PL_{baseline}$)]. All ratios were restricted to regioisomers at the same double bond. The least-squares mean [95% CI] are shown. Note the log scale of the *y*-axis.
