# Supplementary material for: Effect of Omega-3 Fatty Acid Ethyl Esters on the Oxylipin Composition of Lipoproteins in Hypertriglyceridemic, Statin-Treated Subjects
Source: PLoS One. 2014 Nov 13;9(11):e111471. doi: 10.1371/journal.pone.0111471 (PMC4230929; doi:10.1371/journal.pone.0111471)
Supplement: File S2 — Supporting tables. (PDF) [file pone.0111471.s003.pdf]

Supplemental Table 1: Least square mean (nM oxy / mM PL) and [95% CI] with treatment effects [95% CI] (adjusted for age and sex).

Lipo	Chemistry	Parent FA	Regioisomer	Baseline	Final	Difference (% change baseline)	pVal
HDL	Alc	LA	9-HODE	460 (350, 610)	530 (400, 690)	14 (-5, 37)	0.15
HDL	Alc	LA	13-HODE	2020 (1530, 2660)	2320 (1760, 3060)	.	.
LDL	Alc	LA	9-HODE	590 (440, 790)	540 (400, 730)	-6 (-21, 12)	0.46
LDL	Alc	LA	13-HODE	2790 (2080, 3750)	2660 (1980, 3580)	.	.
VLDL	Alc	LA	9-HODE	570 (380, 870)	630 (420, 950)	9 (-19, 46)	0.57
VLDL	Alc	LA	13-HODE	2690 (1780, 4070)	2890 (1910, 4370)	.	.
**Plasma**	**Alc**	**LA**	**9-HODE**	**380 (300, 470)**	**440 (350, 550)**	**15 (2, 29)**	**0.03**
**Plasma**	**Alc**	**LA**	**13-HODE**	**1660 (1330, 2080)**	**1890 (1510, 2360)**	**.**	**.**
HDL	Alc	aLA	9-HOTE	4.1 (3.0, 5.7)	5.0 (3.7, 6.9)	14 (-9, 43)	0.26
HDL	Alc	aLA	13-HOTE	6.1 (4.5, 8.4)	6.5 (4.8, 9.0)	.	.
LDL	Alc	aLA	9-HOTE	5.0 (3.6, 7.1)	5.5 (3.9, 7.8)	4 (-15, 27)	0.72
LDL	Alc	aLA	13-HOTE	4.3 (3.1, 6.1)	4.2 (3.0, 5.9)	.	.
VLDL	Alc	aLA	9-HOTE	18.6 (11.7, 29.5)	22.0 (13.9, 35.0)	15 (-20, 66)	0.43
VLDL	Alc	aLA	13-HOTE	17.2 (10.8, 27.2)	19.2 (12.1, 30.5)	.	.
Plasma	Alc	aLA	9-HOTE	7.8 (5.7, 10.6)	9.1 (6.7, 12.4)	15 (-7, 42)	0.18
Plasma	Alc	aLA	13-HOTE	10.4 (7.6, 14.2)	11.9 (8.7, 16.2)	.	.
HDL	Alc	dgLA	15(S)-HETrE	38 (30, 49)	35 (27, 44)	-10 (-31, 18)	0.41
LDL	Alc	dgLA	15(S)-HETrE	26 (18, 39)	20 (14, 30)	-22 (-47, 16)	0.2
VLDL	Alc	dgLA	15(S)-HETrE	24 (16, 36)	18 (12, 27)	-25 (-59, 36)	0.32
Plasma	Alc	dgLA	15(S)-HETrE	26 (19, 35)	24 (18, 32)	-8 (-34, 29)	0.62
**HDL**	**Alc**	**AA**	**5-HETE**	**208 (162, 267)**	**172 (134, 220)**	**-15 (-24, -4)**	**0.01**
**HDL**	**Alc**	**AA**	**9-HETE**	**168 (131, 216)**	**155 (121, 199)**	**.**	**.**
**HDL**	**Alc**	**AA**	**12-HETE**	**165 (129, 212)**	**135 (105, 173)**	**.**	**.**
**HDL**	**Alc**	**AA**	**15-HETE**	**554 (432, 711)**	**478 (373, 613)**	**.**	**.**
**LDL**	**Alc**	**AA**	**5-HETE**	**162 (125, 211)**	**119 (92, 155)**	**-29 (-36, -21)**	**<0.0001**
**LDL**	**Alc**	**AA**	**9-HETE**	**137 (105, 177)**	**104 (80, 135)**	**.**	**.**
**LDL**	**Alc**	**AA**	**12-HETE**	**104 (80, 134)**	**74 (57, 96)**	**.**	**.**
**LDL**	**Alc**	**AA**	**15-HETE**	**327 (252, 424)**	**211 (163, 274)**	**.**	**.**
**VLDL**	**Alc**	**AA**	**5-HETE**	**112 (72, 175)**	**88 (56, 138)**	**-18 (-32, 0)**	**0.05**
**VLDL**	**Alc**	**AA**	**9-HETE**	**81 (52, 127)**	**69 (44, 107)**	**.**	**.**
**VLDL**	**Alc**	**AA**	**12-HETE**	**54 (34, 84)**	**43 (28, 67)**	**.**	**.**
**VLDL**	**Alc**	**AA**	**15-HETE**	**162 (104, 254)**	**138 (88, 215)**	**.**	**.**
Plasma	Alc	AA	5-HETE	121 (90, 163)	120 (89, 162)	-4 (-17, 10)	0.55
Plasma	Alc	AA	9-HETE	56 (41, 75)	54 (40, 73)	.	.
Plasma	Alc	AA	12-HETE	62 (46, 83)	55 (41, 74)	.	.
Plasma	Alc	AA	15-HETE	310 (231, 417)	304 (226, 410)	.	.
**HDL**	**Alc**	**EPA**	**5-HEPE**	**14.7 (10.6, 20.3)**	**53.1 (38.4, 73.4)**	**294 (212, 397)**	**<0.0001**
**HDL**	**Alc**	**EPA**	**12-HEPE**	**10.3 (7.4, 14.2)**	**42.9 (31.1, 59.3)**	**.**	**.**
**HDL**	**Alc**	**EPA**	**15-HEPE**	**3.6 (2.6, 5)**	**14.6 (10.5, 20.1)**	**.**	**.**
**LDL**	**Alc**	**EPA**	**5-HEPE**	**10.9 (7.8, 15.2)**	**42.2 (30.2, 58.9)**	**303 (232, 391)[a]**	**<0.0001**

**LDL**	**Alc**	**EPA**	**12-HEPE**	**7 (5, 9.8)**	**28.4 (20.4, 39.7)**	**.**	**.**
**LDL**	**Alc**	**EPA**	**15-HEPE**	**2.3 (1.7, 3.3)**	**9.9 (7.1, 13.8)**	**.**	**.**
**VLDL**	**Alc**	**EPA**	**5-HEPE**	**10.2 (6, 17.2)**	**47 (27.8, 79.6)**	**352 (228, 522)** [a]	**<0.0001**
**VLDL**	**Alc**	**EPA**	**12-HEPE**	**5.5 (3.2, 9.2)**	**23.4 (13.9, 39.6)**	**.**	**.**
**VLDL**	**Alc**	**EPA**	**15-HEPE**	**1.8 (1.1, 3.1)**	**8.6 (5.1, 14.5)**	**.**	**.**
**Plasma**	**Alc**	**EPA**	**5-HEPE**	**12.6 (8.9, 17.9)**	**61.7 (43.5, 87.5)**	**400 (313, 505)**	**<0.0001**
**Plasma**	**Alc**	**EPA**	**12-HEPE**	**6.5 (4.6, 9.2)**	**30.6 (21.6, 43.4)**	**.**	**.**
**Plasma**	**Alc**	**EPA**	**15-HEPE**	**2.2 (1.6, 3.2)**	**12.1 (8.6, 17.2)**	**.**	**.**
**HDL**	**Alc**	**DHA**	**17-HDoHE**	**134 (101, 178)**	**276 (208, 367)**	**106 (23, 244)**	**0.009**
**LDL**	**Alc**	**DHA**	**17-HDoHE**	**48 (35, 67)**	**85 (61, 117)**	**75 (24, 149)**	**0.004**
**VLDL**	**Alc**	**DHA**	**17-HDoHE**	**42 (26, 67)**	**135 (84, 215)**	**220 (57, 552)** [a]	**0.003**
**Plasma**	**Alc**	**DHA**	**17-HDoHE**	**71 (50, 102)**	**183 (128, 264)**	**157 (71, 287)**	**0.0002**
HDL	Ket	LA	9-KODE	109 (91, 131)	109 (91, 131)	0 (-15, 17)	0.97
LDL	Ket	LA	9-KODE	191 (153, 238)	181 (145, 226)	-5 (-24, 18)	0.61
VLDL	Ket	LA	9-KODE	284 (197, 408)	319 (221, 459)	12 (-32, 85)	0.63
Plasma	Ket	LA	9-KODE	89 (64, 124)	100 (71, 139)	12 (-13, 44)	0.35
**HDL**	**Ket**	**AA**	**5-KETE**	**38 (27, 53)**	**28 (20, 39)**	**-32 (-45, -17)**	**0.0004**
**HDL**	**Ket**	**AA**	**15-KETE**	**92 (66, 128)**	**57 (41, 79)**	**.**	**.**
**LDL**	**Ket**	**AA**	**5-KETE**	**44 (33, 58)**	**32 (24, 43)**	**-32 (-42, -20)**	**<0.0001**
**LDL**	**Ket**	**AA**	**15-KETE**	**132 (99, 175)**	**82 (62, 110)**	**.**	**.**
VLDL	Ket	AA	5-KETE	45 (27, 75)	38 (23, 63)	-20 (-44, 16)	0.23
VLDL	Ket	AA	15-KETE	245 (148, 407)	188 (114, 313)	.	.
Plasma	Ket	AA	5-KETE	52 (36, 74)	47 (33, 68)	-10 (-30, 15)	0.39
Plasma	Ket	AA	15-KETE	266 (185, 381)	234 (163, 336)	.	.
HDL	Leuko	AA	6-trans-LTB4	.	.	.	.
LDL	Leuko	AA	6-trans-LTB4	0.15 (0.10, 0.20)	0.12 (0.09, 0.17)	-14 (-47, 39)	0.50
VLDL	Leuko	AA	6-trans-LTB4	0.078 (0.045, 0.134)	0.073 (0.043, 0.127)	-6 (-57, 106)	0.88
Plasma	Leuko	AA	6-trans-LTB4	0.078 (0.057, 0.109)	0.075 (0.054, 0.104)	-4 (-33, 37)	0.8
HDL	Epox	LA	9(10)-EpOME	273 (210, 355)	273 (210, 354)	1 (-13, 19)	0.86
HDL	Epox	LA	12(13)-EpOME	328 (253, 426)	338 (260, 438)	.	.
LDL	Epox	LA	9(10)-EpOME	237 (188, 300)	236 (187, 299)	-2 (-13, 11)	0.78
LDL	Epox	LA	12(13)-EpOME	300 (237, 379)	291 (230, 367)	.	.
VLDL	Epox	LA	9(10)-EpOME	269 (194, 374)	277 (199, 385)	5 (-19, 35)	0.71
VLDL	Epox	LA	12(13)-EpOME	324 (233, 451)	347 (250, 482)	.	.
Plasma	Epox	LA	9(10)-EpOME	134 (99, 181)	152 (112, 205)	9 (-10, 33)	0.38
Plasma	Epox	LA	12(13)-EpOME	157 (116, 212)	165 (122, 223)	.	.
HDL	Epox	aLA	9(10)-EpODE	19.4 (12.3, 30.6)	20.5 (13, 32.4)	7 (-18, 40)	0.62
HDL	Epox	aLA	12(13)-EpODE	4.1 (2.6, 6.4)	4.6 (2.9, 7.3)	.	.
HDL	Epox	aLA	15(16)-EpODE	17.6 (11.2, 27.8)	18.1 (11.5, 28.5)	.	.
LDL	Epox	aLA	9(10)-EpODE	15.2 (12.3, 18.7)	14.4 (11.6, 17.8)	-4 (-17, 12)	0.62
LDL	Epox	aLA	12(13)-EpODE	2.1 (1.7, 2.5)	2.1 (1.7, 2.6)	.	.
LDL	Epox	aLA	15(16)-EpODE	16 (12.9, 19.7)	14.8 (11.9, 18.3)	.	.
VLDL	Epox	aLA	9(10)-EpODE	48 (32.5, 70.8)	56.4 (38.2, 83.2)	21 (-4, 51)	0.10
VLDL	Epox	aLA	12(13)-EpODE	6.4 (4.3, 9.4)	8.2 (5.5, 12.1)	.	.

VLDL	Epox	aLA	15(16)-EpODE	50.2 (34, 74.1)	58.4 (39.6, 86.2)	.	.
Plasma	Epox	aLA	9(10)-EpODE	28.2 (20.5, 38.7)	31.5 (22.9, 43.3)	11 (-5, 30)	0.17
Plasma	Epox	aLA	12(13)-EpODE	2.5 (1.8, 3.5)	2.9 (2.1, 4.0)	.	.
Plasma	Epox	aLA	15(16)-EpODE	29.4 (21.4, 40.4)	31.5 (22.9, 43.3)	.	.
**HDL**	**Epox**	**AA**	**8(9)-EpETrE**	**106 (75, 150)**	**77 (54, 109)**	**-23 (-33, -12)**	**0.0004**
**HDL**	**Epox**	**AA**	**11(12)-EpETrE**	**262 (185, 372)**	**207 (146, 293)**	**.**	**.**
**HDL**	**Epox**	**AA**	**14(15)-EpETrE**	**208 (147, 295)**	**165 (116, 234)**	**.**	**.**
**LDL**	**Epox**	**AA**	**8(9)-EpETrE**	**55 (41, 74)**	**39 (29, 53)**	**-27 (-35, -19)**	**<0.0001**
**LDL**	**Epox**	**AA**	**11(12)-EpETrE**	**131 (97, 175)**	**101 (75, 135)**	**.**	**.**
**LDL**	**Epox**	**AA**	**14(15)-EpETrE**	**114 (85, 153)**	**81 (60, 109)**	**.**	**.**
VLDL	Epox	AA	8(9)-EpETrE	39 (25, 60)	35 (23, 54)	-9 (-24, 9)	0.31
VLDL	Epox	AA	11(12)-EpETrE	88 (57, 137)	79 (51, 122)	.	.
VLDL	Epox	AA	14(15)-EpETrE	73 (47, 113)	69 (44, 106)	.	.
Plasma	Epox	AA	8(9)-EpETrE	39 (28, 54)	34 (25, 48)	-8 (-19, 6)	0.24
Plasma	Epox	AA	11(12)-EpETrE	87 (62, 120)	80 (58, 112)	.	.
Plasma	Epox	AA	14(15)-EpETrE	70 (50, 97)	68 (49, 94)	.	.
**HDL**	**Epox**	**EPA**	**14(15)-EpETE**	**10.5 (6.4, 17.2)**	**43.2 (26.8, 69.8)**	**325 (184, 536)**	**<0.0001**
**HDL**	**Epox**	**EPA**	**17(18)-EpETE**	**18.7 (11.7, 29.8)**	**81.7 (51.3, 130.2)**	**.**	**.**
**LDL**	**Epox**	**EPA**	**14(15)-EpETE**	**5.1 (3.5, 7.5)**	**22.7 (15.5, 33.4)**	**318 (224, 439)**	**<0.0001**
**LDL**	**Epox**	**EPA**	**17(18)-EpETE**	**10.4 (7.1, 15.3)**	**41.1 (28, 60.3)**	**.**	**.**
**VLDL**	**Epox**	**EPA**	**14(15)-EpETE**	**5.0 (3.0, 8.5)**	**22.7 (13.4, 38.3)**	**379 (236, 584)**	**<0.0001**
**VLDL**	**Epox**	**EPA**	**17(18)-EpETE**	**6.4 (3.8, 10.8)**	**32.5 (19.2, 54.8)**	**.**	**.**
**Plasma**	**Epox**	**EPA**	**14(15)-EpETE**	**2.4 (1.6, 3.6)**	**13.6 (8.9, 20.8)**	**436 (307, 606)**	**<0.0001**
**Plasma**	**Epox**	**EPA**	**17(18)-EpETE**	**6.0 (3.9, 9.1)**	**29.8 (19.5, 45.5)**	**.**	**.**
**HDL**	**Epox**	**DHA**	**16(17)-EpDPE**	**43 (27, 67)**	**77 (49, 121)**	**81 (7, 206)**	**0.03**
LDL	Epox	DHA	16(17)-EpDPE	18 (12, 26)	29 (19, 43)	63 (-8, 188)	0.09
**VLDL**	**Epox**	**DHA**	**16(17)-EpDPE**	**14 (8, 24)**	**56 (33, 95)**	**304 (107, 690)**	**0.0005**
**Plasma**	**Epox**	**DHA**	**16(17)-EpDPE**	**16 (11, 25)**	**46 (30, 70)**	**182 (73, 358)**	**0.004**
HDL	Diol	LA	9,10-DiHOME	46 (36, 59)	45 (35, 59)	2 (-18, 27)	0.86
HDL	Diol	LA	12,13-DiHOME	8.5 (6.6, 11)	8.9 (6.9, 11.4)	.	.
LDL	Diol	LA	9,10-DiHOME	20 (16, 25)	18 (14, 22)	-4 (-18, 13)	0.63
LDL	Diol	LA	12,13-DiHOME	3.4 (2.8, 4.3)	3.6 (2.9, 4.4)	.	.
VLDL	Diol	LA	9,10-DiHOME	16 (11, 24)	17 (11, 25)	21 (-13, 67)	0.25
VLDL	Diol	LA	12,13-DiHOME	2.3 (1.6, 3.5)	3.2 (2.1, 4.9)	.	.
**Plasma**	**Diol**	**LA**	**9,10-DiHOME**	21 (17, 28)	24 (19, 31)	**24 (1, 52)**	**0.04**
**Plasma**	**Diol**	**LA**	**12,13-DiHOME**	1.6 (1.3, 2.1)	2.3 (1.8, 2.9)	**.**	**.**
HDL	Diol	aLA	9,10-DiHODE	.	.	.	.
LDL	Diol	aLA	9,10-DiHODE	.	.	.	.
VLDL	Diol	aLA	9,10-DiHODE	0.07 (0.04, 0.14)	0.09 (0.04, 0.17)	19 (-54, 207)	0.7
Plasma	Diol	aLA	9,10-DiHODE	0.08 (0.06, 0.13)	0.10 (0.07, 0.16)	22 (-27, 105)	0.41
**HDL**	**Diol**	**AA**	**5,6-DiHETrE**	**3.54 (2.88, 4.36)**	**2.63 (2.14, 3.23)**	**-22 (-31, -13)**	**<0.0001**
**HDL**	**Diol**	**AA**	**8,9-DiHETrE**	**1.89 (1.54, 2.33)**	**1.38 (1.12, 1.69)**	**.**	**.**
**HDL**	**Diol**	**AA**	**11,12-DiHETrE**	**0.60 (0.49, 0.74)**	**0.44 (0.36, 0.55)**	**.**	**.**
**HDL**	**Diol**	**AA**	**14,15-DiHETrE**	**0.50 (0.40, 0.61)**	**0.45 (0.37, 0.55)**	**.**	**.**

**LDL**	**Diol**	**AA**	**5,6-DiHETrE**	**4.31 (3.47, 5.36)**	**3.09 (2.49, 3.84)**	**-28 (-34, -21)**	**<0.0001**
**LDL**	**Diol**	**AA**	**8,9-DiHETrE**	**1.62 (1.30, 2.01)**	**1.12 (0.90, 1.39)**	**.**	**.**
**LDL**	**Diol**	**AA**	**11,12-DiHETrE**	**0.55 (0.44, 0.68)**	**0.38 (0.30, 0.47)**	**.**	**.**
**LDL**	**Diol**	**AA**	**14,15-DiHETrE**	**0.38 (0.31, 0.48)**	**0.30 (0.24, 0.38)**	**.**	**.**
**VLDL**	**Diol**	**AA**	**5,6-DiHETrE**	**2.66 (1.67, 4.22)**	**2.20 (1.39, 3.49)**	**-19 (-34, -2)**	**0.03**
**VLDL**	**Diol**	**AA**	**8,9-DiHETrE**	**1.10 (0.69, 1.74)**	**0.79 (0.50, 1.26)**	**.**	**.**
**VLDL**	**Diol**	**AA**	**11,12-DiHETrE**	**0.37 (0.23, 0.58)**	**0.28 (0.18, 0.45)**	**.**	**.**
**VLDL**	**Diol**	**AA**	**14,15-DiHETrE**	**0.27 (0.17, 0.43)**	**0.24 (0.15, 0.39)**	**.**	**.**
Plasma	Diol	AA	5,6-DiHETrE	2.93 (2.33, 3.68)	2.51 (2.00, 3.15)	-7 (-15, 2)	0.12
Plasma	Diol	AA	8,9-DiHETrE	1.17 (0.93, 1.47)	1.10 (0.88, 1.39)	.	.
Plasma	Diol	AA	11,12-DiHETrE	0.37 (0.29, 0.46)	0.32 (0.26, 0.41)	.	.
Plasma	Diol	AA	14,15-DiHETrE	0.24 (0.19, 0.31)	0.25 (0.20, 0.32)	.	.
**HDL**	**Diol**	**EPA**	**14,15-DiHETE**	**1.01 (0.69, 1.48)**	**1.85 (1.33, 2.57)**	**49 (2, 118)**	**0.04**
**HDL**	**Diol**	**EPA**	**17,18-DiHETE**	**4.26 (2.99, 6.08)**	**5.17 (3.64, 7.34)**	**.**	**.**
**LDL**	**Diol**	**EPA**	**14,15-DiHETE**	**0.41 (0.27, 0.61)**	**0.83 (0.56, 1.24)**	**97 (46, 166)**	**<0.0001**
**LDL**	**Diol**	**EPA**	**17,18-DiHETE**	**0.60 (0.38, 0.95)**	**1.13 (0.75, 1.70)**	**.**	**.**
**VLDL**	**Diol**	**EPA**	**14,15-DiHETE**	**0.33 (0.22, 0.50)**	**1.08 (0.71, 1.63)**	**109 (47, 196)**	**0.0001**
**VLDL**	**Diol**	**EPA**	**17,18-DiHETE**	**0.68 (0.45, 1.05)**	**0.90 (0.59, 1.36)**	**.**	**.**
**Plasma**	**Diol**	**EPA**	**14,15-DiHETE**	**0.22 (0.15, 0.32)**	**0.68 (0.47, 0.98)**	**158 (99, 235)**	**<0.0001**
**Plasma**	**Diol**	**EPA**	**17,18-DiHETE**	**0.31 (0.21, 0.45)**	**0.64 (0.44, 0.94)**	**.**	**.**
HDL	Diol	DHA	19,20-DiHDPA	0.36 (0.24, 0.56)	0.33 (0.21, 0.51)	-9 (-44, 49)	0.70
**LDL**	**Diol**	**DHA**	**19,20-DiHDPA**	**0.06 (0.04, 0.09)**	**0.12 (0.08, 0.17)**	**104 (55, 168)**	**0.0001**
**VLDL**	**Diol**	**DHA**	**19,20-DiHDPA**	**0.08 (0.05, 0.13)**	**0.22 (0.14, 0.37)**	**180 (44, 443)**	**0.01**
**Plasma**	**Diol**	**DHA**	**19,20-DiHDPA**	**0.06 (0.04, 0.08)**	**0.16 (0.12, 0.22)**	**162 (92, 258)**	**<0.0001**
HDL	PG	AA	PGF2a	.	.	.	.
LDL	PG	AA	PGF2a	0.35 (0.20, 0.60)	0.34 (0.20, 0.58)	-2 (-40, 59)	0.91
VLDL	PG	AA	PGF2a	0.69 (0.44, 1.07)	0.45 (0.28, 0.71)	-35 (-64, 17)	0.14
Plasma	PG	AA	PGF2a	0.84 (0.58, 1.21)	0.59 (0.40, 0.85)	-30 (-53, 4)	0.07
HDL	Triol	LA	9,10-13-TriHOME	5.6 (3.3, 9.5)	5.9 (3.6, 9.6)	19 (-18, 73)	0.34
HDL	Triol	LA	9,12,13-TriHOME	13.6 (8.6, 21.5)	17.8 (11.4, 27.8)	.	.
LDL	Triol	LA	9,10-13-TriHOME	1.5 (0.9, 2.4)	1.7 (1.1, 2.7)	11 (-17, 48)	0.49
LDL	Triol	LA	9,12,13-TriHOME	5.0 (3.1, 8.1)	5.4 (3.4, 8.6)	.	.
VLDL	Triol	LA	9,10-13-TriHOME	1.7 (1.2, 2.5)	1.9 (1.3, 2.8)	8 (-21, 48)	0.62
VLDL	Triol	LA	9,12,13-TriHOME	5.4 (3.7, 7.9)	5.7 (3.9, 8.3)	.	.
Plasma	Triol	LA	9,10-13-TriHOME	1.0 (0.7, 1.5)	1.2 (0.9, 1.8)	16 (-6, 44)	0.17
Plasma	Triol	LA	9,12,13-TriHOME	3.1 (2.2, 4.5)	3.5 (2.5, 5.0)	.	.
HDL	EpKet	LA	EKODE	57 (45, 71)	58 (46, 73)	2 (-21, 31)	0.87
LDL	EpKet	LA	EKODE	92 (72, 117)	86 (68, 110)	-6 (-25, 17)	0.56
VLDL	EpKet	LA	EKODE	126 (88, 183)	139 (96, 201)	10 (-33, 81)	0.69
Plasma	EpKet	LA	EKODE	50 (39, 64)	53 (41, 68)	6 (-19, 39)	0.63

[a] The adjustment for age was significant (P<0.05).

Supplmental Table 2: Correlation between oxylipins and erythrocyte content of parent fatty acid.

Oxylipin	Visit	HDL	LDL	VLDL	Plasma	Oxylipin	HDL	LDL	VLDL	Plasma
		Pearson Correlation					Pearson Correlation			
		**Mid-chain alcohols**					**Ketones**			
		Arachidonates								
5-HETE	*Baseline*	**0.79**				5-KETE				
	Final									
9-HETE	*Baseline*	**0.58**								
	Final									
12-HETE	*Baseline*									
	Final									
15-HETE	*Baseline*	**0.69**				15-KETE				
	Final									
		Eicosapentaenoates								
5-HEPE	*Baseline*		**0.67**	**0.80**	**0.85**					
	Final	**0.88**	**0.58**	**0.59**	**0.81**					
12-HEPE	*Baseline*	**0.53**	**0.63**	**0.72**	**0.63**					
	Final	**0.85**	**0.68**	**0.72**	**0.81**					
15-HEPE	*Baseline*	**0.64**	**0.57**	**0.72**	**0.74**					
	Final	**0.82**	**0.60**	**0.69**	**0.77**					
		Docosahexaenoates								
17-HDoHE	*Baseline*		**0.60**		**0.64**					
	Final	**0.63**								
		**Epoxides**					**Vicinal Diols**			
		Arachidonates								
8(9)-EpETrE	*Baseline*					8,9-DiHETrE				
	Final									
11(12)-EpETrE	*Baseline*					11,12-DiHETrE				
	Final									
14(15)-EpETrE	*Baseline*					14,15-DiHETrE				
	Final									
		Eicosapentaenoates								
14(15)-EpETE	*Baseline*	**0.63**	**0.78**	**0.63**	**0.56**	14,15-DiHETE			**0.80**	
	Final	**0.58**	**0.70**	**0.78**	**0.76**		**0.65**	**0.63**		**0.88**
17(18)-EpETE	*Baseline*	**0.58**	**0.81**	**0.66**	**0.70**	17,18-DiHETE			**0.65**	
	Final	**0.64**	**0.71**	**0.83**	**0.81**				**0.69**	**0.85**
		Docosahexaenoates								
16(17)-EpDPE	*Baseline*		**0.81**	**0.66**	**0.73**	19,20-DiHDPA				**0.61**

Pearson correlation on log(nM oxylipin) × log(erythrocyte parent FA as %total)

Where the regression coefficient is significant at $p<0.05$, it is represented in **bold**; where $p>0.05$, only color is represented.

Supplemental Table 2; Key

Key	
r	Color
1.00	
0.67	
0.33	
0.00	
-0.33	
-0.67	
-1.00	

Supplemental Table 3: Estimation of changes in epoxide bioactivity with concentrations unmeasured epoxides imputed[a]

	Net potency for vasodilation							
	**HDL**		**LDL**		**VLDL**		**Plasma**	
	Bs	***Final***	***Bs***	***Final***	***Bs***	***Final***	***Bs***	***Final***
**Net AA** [32]	0.99	0.77	0.52	0.38	0.34	0.31	0.33	0.31
**Net EPA** [32]	0.21	0.85	0.11	0.44	0.09	0.41	0.06	0.28
**Net DHA** [31]	0.21	0.39	0.09	0.15	0.07	0.29	0.08	0.23
**Total**	**1.41**	**2.02**	**0.71**	**0.96**	**0.50**	**1.00**	**0.47**	**0.82**
**Fold-change**		**1.4**		**1.4**		**2.0**		**1.7**

[a] imputations were made using the reactivity of each double bond implied by Newman and Hammock [33].

[b] Net potency measured as Σ -EC_{50} × [*Epoxide*]$_{measured\ or\ imputed}$ as reported by Ye *et al* & Zhang *et al*.

Note: potencies are measured in nM^2 / mM PL. Abbreviation: Bs – Baseline.
