# Supplementary material for: Effect of Omega-3 Fatty Acid Ethyl Esters on the Oxylipin Composition of Lipoproteins in Hypertriglyceridemic, Statin-Treated Subjects
Source: PLoS One. 2014 Nov 13;9(11):e111471. doi: 10.1371/journal.pone.0111471 (PMC4230929; doi:10.1371/journal.pone.0111471)
Supplement: File S3 — Supplemental list of abbreviations. (DOCX) [file pone.0111471.s004.docx]

**Summary of Abbreviations & Terms**

| 18:2n6 | linoleic acid |
| --- | --- |
| 18:3n3 | alpha-linolenic acid |
| 18:3n6 | gamma-linolenic acid |
| 20:3n6 | dihomo-γ-linolenic acid |
| 20:4n6 | arachidonic acid |
| 20:5n3 | eicosapentaenoic acid |
| 22:6n3 | docosahexaenoic acid |
| 6-trans-LTB4 | 6-trans leukotriene b4 |
| AA | arachidonic acid |
| aLA | α-linolenic acid |
| BMI | body mass index |
| BPM | beats per minute |
| COMBOS | Combination of Prescription Omega-3 Plus Simvastatin Trial |
| COX | cyclooxygenase |
| CVD | cardiovascular disease |
| CYP | cytochrome p450 |
| DHA | docosahexaenoic acid |
| DiHDPA | dihydroxydocosapentaenoic acid |
| DiHETE | dihydroxyeicosatetraenoic acid |
| DiHETrE | dihydroxyeicosatrienoic acid |
| DiHODE | dihydroxyoctadecadienoic acid |
| DiHOME | dihydroxyoctadecamonoenoic acid |
| EKODE | epoxyketooctadecenoic acid |
| EPA | eicosapentaenoic acid |
| EpDPE | epoxyedocosapentaenoic acid |
| EpETE | epoxyeicosatetraenoic acid |
| EpETrE | epoxyeicosatrienoic acid |
| EpODE | epoxyoctadecadienoic acid |
| EpOME | epoxyoctadecamonoenoic acid |
| FA | fatty acid |
| FPLC | fast protein liquid chromatography |
| HDL | High Density Lipoprotein |
| HDoHE | hydroxydocosahexaenoic acid |
| HEPE | hydroxyeicosapentaenoic acid |
| HETE | hydroxyeicosatetraenoic acid |
| HETrE | hydroxyeicosatrienoic acid |
| HODE | hydroxyoctadecadienoic acid |
| HOTE | hydroxyoctadecatrienoic acid |
| IND | investigational new drug |
| IRB | institutional review board |
| KETE | ketoeicosatetraenoic acid |
| KODE | ketooctadecadienoic acid |
| LA | linoleic acid |
| LDL | Low Density Lipoprotein |
| LOX | lipoxygenase |
| NCEP | National Cholesterol Education Program |
| OMX-3 | omega-3 index |
| PBS | phosphate buffered saline |
| PGF2a | prostaglandin f2 alpha |
| PL | phospholipid |
| P‑OM3 | prescription omega-3 |
| PPAR | peroxisome proliferator-activated receptor |
| RBC | red blood cell |
| TG | triglyceride |
| TriHOME | trihydroxyoctadecamonoenoic acid |
| VLDL | Very Low Density Lipoprotein |
