# Supplementary material for: Effect of Omega-3 Fatty Acid Ethyl Esters on the Oxylipin Composition of Lipoproteins in Hypertriglyceridemic, Statin-Treated Subjects
Source: PLoS One. 2014 Nov 13;9(11):e111471. doi: 10.1371/journal.pone.0111471 (PMC4230929; doi:10.1371/journal.pone.0111471)
Supplement: Protocol S1 — Study protocol. (PDF) [file pone.0111471.s005.pdf]

Effects of Lovaza on Lipoprotein composition and Function in mild hypertriglyceridemia (ELLF)

Gregory Shearer, PhD[1] William Harris, PhD[1] and Verdayne Brandenburg, MD

[1]Sanford Research/USD, [2]Sanford Clinic, Sioux Falls, SD

Overview

This study will build upon a previous finding that showed a triglyceride lowering effect of prescription omega-3 in combined therapy with statins. The proposed study will use a simple change from baseline design on 15 subjects who are hypertriglyceridemic on stable statin therapy. The protocol involves 3 study visits; each involve drawing a blood sample: day 1 – screening (blood draw for qualification and safety); day 7 – before taking prescription omega-3 fatty acids (Lovaza, 4 capsules/d; GlaxoSmithKline), day 63 –after taking prescription omega-3 fatty acids (Lovaza, 4 capsules/d; GlaxoSmithKline). The proposed study will include up to 15 subjects and will utilize several different measures of lipoprotein structure and function. We will measure fatty acid, oxylipin and apolipoprotein content of plasma, VLDL, LDL and HDL. We will also measure functional parameters such as the binding affinity of lipoproteins before and after treatment with omega-3 fatty acids. These studies will provide the most in depth examination yet of the single and combined effects of prescription omega-3 and statins on lipoprotein function, and will indicate the specific mechanisms by which omega-3 fatty acids alter (or do not alter) human lipoprotein clearance in the context of hypertriglyceridemia and statin therapy.

Background/Significance

Lovaza is a pharmaceutical-grade omega-3 fatty acid product providing 465 mg eicosapentaenoic acid (EPA) and 375 mg docosahexaenoic acid (DHA) in a 1-g gel capsule. It is currently approved by the FDA for treatment of severe hypertriglyceridemia (≥500 mg/dL), and is likely to soon be approved for the treatment of moderate hypertriglyceridemia (200 < triglyceride <500 mg/dL) in combination with statins. In other words, the use of Lovaza will continue to expand. Increased clearance of VLDL is a mechanism by which Lovaza decreases serum triglycerides, but it is not known how clearance is increased. Prior studies on the effects of fish oils indicate that both improved lipoprotein binding and better performance as a substrate for lipoprotein lipase may be involved. This study will address these unknowns.

Experimental Design and Methodology

Design

This will be an open-label, baseline vs. end of treatment, 8-week trial.

Subjects

Fifteen volunteers with mild hypertriglyceridemia (between 200 and 499 mg/dL) while on stable statin therapy will be recruited from among the patient population of Sanford Clinic. Subjects may be male or female. The following inclusion/exclusion criteria will apply:

<u>Inclusion</u>

1. 18-79 yrs
2. Stable does of statins ≥ 8 weeks prior to screening
3. Good health by medical history, physical exam, electrocardiogram, laboratory test (e.g. serum chem., urinalysis)
4. Mean fasting serum triglyceride of two most recent tests in medical record ≥ 200 and < 500 mg/dL

5. Mean LDL-cholesterol of two most recent tests in medical record ≤ 1.1 × NCEP ATP III goal

Exclusion

1. Medications, vitamin pills, nutritional supplements or herbal preparations deemed exclusionary per primary investigator for possible interference
2. Poorly controlled diabetes mellitus (*e.g.* [HbA_{1c}] > 8.0%)
3. History of a cardiovascular event
4. Past revascularization procedure
5. Past aortic aneurysm or an aortic dissection < 6 months prior to screening
6. History of pancreatitis
7. Sensitivity to any statin OR to omega-3 fatty acids or fish products
8. Poorly controlled hypertension
   a. ≥ 160 systolic (resting)
   and/or
   b. ≥ 100 diastolic (resting)
   at 2 consecutive visits
9. Serum Creatinine ≥ 2.0 mg/dL
10. Serum transaminase > 1.5 × upper limit of normal (ULN); including aspartate aminotransferase [AST] or alanine aminotransferase [ALT]; 31 U/L for AST, 45 U/L for ALT
11. Creatine Kinase (CK) > 3.0 × ULN
12. Taking other triglyceride lowering drugs (*e.g.* niacin, fibrates) or fish oil supplements providing more than 500 mg of EPA+DHA per day
13. Contraindications for Lovaza per product insert
14. Women who are pregnant or nursing

Other exclusions include: drinking more than two alcoholic beverages a day for males or more than 1 alcoholic beverage per day for females, consuming tuna or other non-fried fish more than 3 times per month, smoking, or having any of the following conditions: an ulcer or bleeding in the stomach, liver or kidney disease, bleeding or blood clotting disorder (*e.g.*, hemophilia), congestive heart failure, heart disease, gout, asthma, arthritis, or nasal polyps.

Protocol

Potential subjects will be screened from the Sanford Health system using Doc-Z. We will contact various primary care physicians within the Sanford Healthcare System. We will give them a presentation about the study and invite them to be involved. If the primary care physician grants us permission to screen their patients, we will then use Doc-Z to conduct a query to identify patients on stable statin therapy with 2 or more measures of serum triglycerides between 200 and 500 mg/dL. Based on our inclusion and exclusion parameters we would also figure out the patient's Framingham Risk Score. In order to do this calculation, we would identify the patient's age, sex, smoking habits, diabetic status, blood pressure, HDL, and the two most recent measures of LDL and triglycerides. We would also use Doc-Z to identify if the patient has any prior history of a past cardiovascular event, revascularization procedure, or if they are on any lipid lowering medications as these are all exclusionary criteria. It is imperative that we use the Doc-Z system in order to gain knowledge of these parameters and in particular in order to gain knowledge of the patient's most recent two measures of triglyceride levels as this greatly improves our statistical power by ensuring that the subjects truly have the triglyceride levels we are targeting in this study. We will then notify their primary care physicians about their possible eligibility in the study. Those subjects will be contacted by a signed letter through the mail from their primary care physician inviting them to participate in the study. If interested, the possible subject will then call or e-mail the Center for Omega-3 research with their contact information. The possible subject will then be

contacted by telephone with more study information and will be invited to answer questions from a phone screen interview questionnaire. If the possible subject chooses not to respond to the letter or does not meet the study criteria following the phone screen, all of the data received from the Doc-Z inquiry will be destroyed. If the subject is qualified following the telephone screening questionnaire, they will be invited to Visit 0 (screening visit) at the Center for Omega-3 Research on the Sanford USD Medical Center Campus. Recruitment advertisements may also be posted in the Sanford Clinics. At the screening visit, the potential subject will provide informed consent and a blood sample will be drawn for assessment of inclusion/exclusion criteria. A short medical history will also be taken. Dr. Brandenburg will review the history and laboratory findings, and determine if the subject is eligible for the study. If so, the subject will return one week later for Study Visit 1 at Dr. Brandenburg's office where a brief physical exam will be conducted. A baseline blood sample will be drawn and the subject will be given an 8-week supply of Lovaza. S/he will be instructed to take 4 capsules per day (2 capsules b.i.d. with meals or all 4 at bedtime), and instructions to follow a heart-healthy diet. Women of childbearing age will also be asked to take a urine pregnancy test. At Study Visit 2, after 8 weeks on study, the subject will return for a final blood draw, a brief review of medical history, and to return any remaining Lovaza. A light breakfast will be available at Visits 1 and 2 following completion of the blood draw.

Visit	Treatment	Day	Procedures
0	Screen	1	Sign informed consent,<br>Overnight fast, 14 mL of blood drawn
1	Baseline<br>(statin only)	7	Overnight fast, 60 mL of blood drawn<br>Begin Lovaza treatment (4 capsules/d)
2	Lovaza<br>(+ statin)	64	Overnight fast, 74 mL of blood drawn<br>Return unused Lovaza<br>Finish

Primary and Secondary endpoints:
The primary endpoint will be the effect of treatment on lipoprotein chemical composition and on VLDL binding to vascular endothelial cells.

Secondary endpoints include:
Performance of VLDL as a substrate for lipolysis.
Plasma LpL mass and activity.
VLDL, LDL, HDL oxylipin fatty acid content.

Laboratory Methods
Blood Samples collected at Visit 0 (Screening Visit) and Visit 2 (Study completion):

<u>14mL Blood Draw for Safety Screening</u>.

2— 5mL gold top tubes for the analysis of Lipid Panel 2, CPK (Total), and Complete Metabolic Panel.
1— 4mL lavender top tube for Hemoglobin A1C analysis.

Blood Samples collected at Study Visits 1 and 2:

EDTA Lavendar tops: 6 tubes @ 10 mL each = 60 mL blood,

RBCs: Keep 0.5 mL for fatty acid analysis

~30 mL plasma: Aliquot as follows:

7—1 mL aliquots, freeze for clinical chemistry (VLDL1,VLDL2,VLDL3,IDL, LDL, HDL2, HDL3)

1—14 mL + sucrose (20%), freeze

1— 3mL for lipoproteins, freeze

1— 1mL for VAP analysis

1— 1mL + sucrose for VAP analysis

Safety

Safety will be monitored by our study physician through medical exam and laboratory tests including Hemoglobin A1C, CPK (total), Complete Metabolic Panel, and Lipid Panel 2 at both the screening visit and upon study completion. Subjects will be told to notify the study coordinator or the PI by telephone if they notice any unusual symptoms of any kind during the 2-month study.

Confidentiality of Data

The identifiers used in this study (name, age, sex, race, weight, height, contact information) will be linked to a unique numerical identifier. The code will be kept in the principal investigator's files (hard copy in a locked file cabinet and electronic copy in a password protected file).

Statistical Approach and Power Calculations

It is a paired design with each subject serving as his/her own control for all assays. Based on an expected standard deviation of 0.2 µg lipoprotein/mg cell protein, we expect to be able to detect a 0.23 µg/mg protein change in binding to vascular endothelial cells with 90% power at $\alpha = 0.01$. For comparison, lipoprotein binding was 0.6 µg/mg protein lower in nephrotic rats (triglycerides = 520 mg/dL) than in control rats (triglycerides = 90 mg/dL). Obviously, we must power for smaller changes since the inclusion criterion require lower initial triglyceride concentrations.

The clinical portion of the study is planned to begin June 9 and to end Dec 2009. Results should be available by December 2009, and a manuscript by March 2010.
